# QTL mapping of vernalization-insensitive and photoperiod-independent early flowering in yellow lupin (*Lupinus luteus* L.)

**DOI:** 10.3389/fpls.2026.1829334

**Published:** 2026-04-23

**Authors:** Ishani Dogra, Allen Eldho-Paul, Anna Surma, Sandra Rychel-Bielska, Wojciech Bielski, Bartosz Kozak, Michał Książkiewicz

**Affiliations:** 1 Department of Gene Structure and Function, Institute of Plant Genetics, Polish Academy of Sciences, Poznań, Poland; 2 Department of Genetics, Plant Breeding and Seed Production, Wroclaw University of Environmental and Life Sciences, Wrocław, Poland

**Keywords:** flowering, genome, linkage map, markers, photoperiod, vernalization

## Abstract

Recent decades have seen noticeable warming, causing in mid and high latitudes longer growing season, more summer droughts, and decreased spring vernalization, prompting plant breeders to focus on improving plant adaptation to these new conditions. One of the key traits is responsiveness to vernalization, a typical characteristic of temperate grain legumes, including yellow lupin (*Lupinus luteus* L.), a potential European alternative to soybeans for sandy soils. Although beneficial for autumn sowing in warmer areas, vernalization responsiveness can be unfavorable for spring sowing due to delayed flowering. To facilitate studies on genetics of vernalization responsiveness in yellow lupin, a recombinant inbred line (RIL) population was developed from a cross between a thermoneutral, rapid flowering breeding line PRH444/14 and a vernalization-responsive, moderately late flowering cultivar, Parys. The RILs were phenotyped in a greenhouse under ambient long-day photoperiod across three spring seasons (2022-2024) and short-day photoperiod during the winter season (2023/4). The difference in flowering time between early and late RILs reached ~39 days in long days and ~68 days in short days. Following DArT-seq genotyping, a linkage map comprising 1,448 loci and anchoring all yellow lupin pseudochromosomes was constructed. Despite moderate fragmentation of the map (63 linkage groups versus 26 chromosomes), there was a high level of collinearity. Composite interval mapping of flowering-related traits identified a major quantitative trait locus (QTL), significant throughout all years and in both photoperiods, located on chromosome YL-16 and explaining up to 34% of the phenotypic variance. This QTL co-localized with a *LlutFTc1* gene, one of the four *Flowering locus T* (*FT*) homologues from the yellow lupin genome. The marker developed for the 2,227 bp indel from the *LlutFTc1* promoter exhibited the highest correlation with plant phenology among all markers on this linkage map. Additionally, several minor QTLs were identified on other chromosomes, including one on chromosome YL-04, significant under long-day photoperiod and explaining up to 5% of phenotypic variance, and another on chromosome YL-14, specific to the short-day photoperiod, accounting for up to 9%. Candidate genes for these QTLs include known flowering time regulators, such as *EAF1B-like* and *CIB2-like* genes for YL-04, and *bHLH93-like* for YL-14.

## 1 Introduction

Yellow lupin (*Lupinus luteus* L.) is a grain legume appreciated for its high protein content (up to 41%) and moderate oil content (up to 11%) in seeds, a remarkable level of mineral elements, and significant antioxidant activity (König, 1883; v. Sengbusch, 1942; Spina et al., 2022). In the context of agronomy and organic farming, the cultivation of yellow lupin is beneficial for soil fertility due to its effective symbiotic nitrogen fixation and has a long history of being used as green manure (Seliga, 1993; Prusinski, 2014a; b; Pietrzykowski et al., 2017). During the 1920s and 1930s, yellow lupin underwent the first round of domestication, which targeted several important agronomic traits, including non-shattering pods, permeable seed coat (soft seediness), and low alkaloid content (v. Sengbuch and Loschakowa, 1932; Hackbarth and v. Sengbusch, 1934; v. Sengbusch, 1934; v. Sengbusch and Zimmermann, 1937; v. Sengbusch, 1938; 1947; Hondelmann, 1984). Further significant improvements included the identification of yellow lupin germplasm resistant to *Fusarium* and anthracnose (v. Sengbusch, 1953; Lamberts, 1955; Adhikari et al., 2011; Lichtin et al., 2020).

Early surveys of the geographical distribution of yellow lupin revealed the abundant presence of landraces and wild populations in Portugal, Spain, Morocco, and Algeria, with some extent into southern Italy, highlighting the area of Northwestern Africa and the Iberian Peninsula as the natural habitat for this species (Fischer and v. Sengbusch, 1935b; Klinkowski, 1938). Following preliminary domestication, the yellow lupin cultivation zone quickly expanded to Central and Eastern Europe, Australia, and New Zealand (Fischer and v. Sengbusch, 1935a; c; Porter and Gilmore, 1976; Cowling and Gladstones, 2000). As the yellow lupin has probably evolved in a relatively dry climate, some intrinsic level of drought tolerance should be present in particular populations. Indeed, a landrace Morocco-4 revealed significant drought tolerance, putatively associated with polyamine deposition (Juzoń et al., 2013, 2017). A substantial increase of summer droughts is currently observed in Europe, with non-negligible contributions from human-driven global climate change (Markonis et al., 2021; Suarez-Gutierrez et al., 2023; Bevacqua et al., 2024), posing a new challenge to breeders. One of the possible solutions is to exploit the drought-escape strategy through early phenology, as tentatively demonstrated for yellow lupin in Australia (Berger and Ludwig, 2014).

Yellow lupin is a plant that responds naturally to vernalization, whereas earliness, which was identified during domestication, was characterized as an abolition of vernalization requirements (Adhikari et al., 2012; Lichtin et al., 2020). To allow efficient cultivation of yellow lupin in northern locations, European breeders aligned its phenology with the duration of the growing season by selecting early flowering germplasm, which is now reflected in the geographic distribution of spring and autumn/winter ecotypes in Europe (Berger et al., 2008). Ongoing global climate change has already resulted in a northward shift of agroclimate zones in Europe in the last 50 years, and this trend is expected to accelerate (King et al., 2018; Ceglar et al., 2019; Akritidis et al., 2023). Yellow lupin breeders responded to this change by developing adapted cultivars. Thus, in the 1930s Poland was localized at the northern edge of the lupin cultivation area (Fischer and v. Sengbusch, 1935a), whereas now Poland has the highest number of yellow lupin cultivars in the Common Catalogue of Varieties of Agricultural Plant Species in the European Union, significantly superseding the countries native to the yellow lupin, such as Portugal (by fivefold) and Spain (by tenfold). To facilitate studies on genetics of flowering time regulation in yellow lupin, a genetic linkage map was developed for a recombinant inbred line (RIL) population descending from a cross between a late flowering P28213 landrace and a moderately early flowering Australian cultivar Wodjil (Iqbal et al., 2019). Further studies based on this RIL population highlighted that flowering time in this mapping population is controlled by several quantitative trait loci (QTLs), localized in genome regions carrying three copies of *Flowering Locus T* (*FT*) gene: one from the clade *FTa* (*LlutFTa1*) and two from the clade *FTc* (*LlutFTc1* and *LlutFTc2*) (Plewiński et al., 2022).

Recently, Polish breeding efforts resulted in the selection of a fully thermoneutral and rapid-flowering yellow lupin line PRH444/14 (COBORU, 2016), advancing by earliness all other lines from the publicly available germplasm (Plewiński et al., 2022). Therefore, in the present study, our objective was to analyze genetic components that contribute to the early phenology of this line. First, the PRH444/14 line was crossed with a moderately late flowering Parys cultivar to generate RIL population segregating for flowering time. Subsequently, this population was advanced to generation F_6_ and was phenotyped in controlled environment for three seasons under a long-day photoperiod (F_6_-F_8_, 2022-2024) and for one season under a short-day photoperiod (F_7_, 2023). DNA isolated from F_8_ plants was submitted for Diversity Arrays Technology sequencing, providing the set of DArT-seq markers for genetic linkage analysis. In parallel, *FT* indel sequences were transformed into PCR markers and incorporated, together with DArT-seq markers, for linkage map development and QTL mapping. Finally, the newly constructed linkage map was aligned with the recently published yellow lupin genome assembly (Martinez-Hernandez et al., 2025) to validate the collinearity between linkage groups and pseudochromosomes, as well as to highlight candidate genes localized in newly identified QTLs.

## 2 Materials and methods

### 2.1 Plant material

The yellow lupin RIL population was developed by Plant Breeding Smolice, Department in Przebędowo, Western Poland (HR Smolice) from a cross between a thermoneutral, rapid flowering breeding line ♂PRH444/14 (paternal) and a vernalization-responsive cultivar ♀Parys (maternal) showing flowering time intermediating between early and late yellow lupin germplasm accessions (Plewiński et al., 2022). Phenotyping was performed for 276 F_6_, F_7_ and F_8_ RILs, while DNA was isolated from F_8_ RILs. Genotyping was performed on F8 generation to keep the level of heterozygosity as low as possible. To keep consistency in genotype-phenotype correlations across generations, RIL population was developed by single seed descent (SSD) with fixed RIL numbering.

### 2.2 Plant phenotyping

Plant phenotyping was carried out in a greenhouse located at the Institute of Plant Genetics, Polish Academy of Sciences, Poznań, Poland (52°26′ N, 16°54′ E) in Poznań (western Poland) under ambient long-day photoperiod reached by spring sowing during three seasons (14^th^ March 2022, 17^th^ March 2023 and 27^th^ March 2024) and under short-day photoperiod realized by autumn sowing during one season (10^th^ November 2023). No pre-sowing vernalization was provided and automatic heating in the greenhouse was set up to keep the minimum air temperature above 18 °C, significantly above the lupin vernalization threshold, which is about 10 °C (Clapham and Willcott, 1995). After spring sowing, the photoperiod was naturally increasing during plant cultivation, starting from about 12 hours to more than 16 hours at the flowering time of late RILs, while after autumn sowing the photoperiod was in the range between 8 and 11 hours. As vernalization-responsive lines considerably delay flowering under non-inductive (short) photoperiod in the absence of vernalization (Christiansen and Jørnsgård, 2002), autumn sowing date was selected for November to induce flowering of such RILs by spring-driven increase of natural photoperiod (to provide complete dataset for QTL mapping).

The plant phenology was inspected every second day. Three flowering-related traits were observed: floral bud emergence (counted as days from sowing to the first bud appearance), start of flowering (recorded when the first fully colored petal was observed), and end of flowering (recorded when the majority of petals on the main stem faded). At least ten biological replicates were sown for each RIL and data for the earliest five plants (in the context of studied traits) were recorded (**Supplementary Table S1**) and used for mean values calculations (**Supplementary Table S2**).

### 2.3 Correlation and heritability calculations

A Kolmogorov-Smirnov test was performed to assess the normality of mean values of phenotypic data (Massey et al., 1951). Then, a calculation of the Spearman rank correlation (Spearman, 1904) was performed for all trait pair combinations. The ranks of the phenotypic observations were determined by using the formula =RANK.AVG in Microsoft Excel. To determine whether the correlation coefficient obtained was statistically significant, a two-tailed *t*-test with a Bonferroni correction applied by multiplying each calculated p-value by the number of analyzed traits was performed. Kruskal Wallis test by ranks with *post-hoc* Dunn’s test was used for inter-annual comparisons (Kruskal and Wallis, 1952; Dunn, 1964). The variance components for the estimation of heritability were obtained by fitting linear mixed models (LMMs) using the ASReml-R package (Butler et al., 2017). In these models, environments (years) were treated as fixed effects, while genotypes and genotype-by-environment interactions were treated as random effects. A heterogeneous residual variance structure was specified in all environments. Broad-sense heritability on a mean basis was calculated from the estimated variance components as *H*^2^ = *σ*^2g^/(*σ*^2g^ + *σ*^2gxe^/*k* + *σ̅*^2e^/(*k* · *r*)), where *σ*^2g^ is the genotypic variance, *σ*^2gxe^ is the genotype-by-environment interaction variance, *σ̅*^2e^ is the mean residual variance across *k* years, and *r* is the average number of replicates per genotype in each environment. Additionally, heritability for data analyzed by LMMs for the best linear unbiased estimators (BLUEs) was calculated using the Cullis method (Cullis et al., 2006) and for the best linear unbiased predictions (BLUPs) using the Piepho method (Piepho and Möhring, 2007), leveraging the average squared standard errors of the differences.

### 2.4 DNA isolation

Two young upper leaves (approximately 50 to 100 mg of tissue per collection tube) were collected from young (five-week-old) single RIL plants and immediately frozen in liquid nitrogen. The frozen plant tissue was homogenized for 45 seconds at 30 rpm using TissueLyser II (Qiagen, Hilden, Germany) and two stainless steel beads (5 mm, Qiagen) placed in 2 mL tubes (Eppendorf, Hamburg, Germany). DNA isolation was performed with the aid of an automated Maxwell^®^ RSC 48 Instrument station (Promega, Mannheim, Germany), using a Maxwell^®^ RSC PureFood GMO and Authentication Kit (Promega) and a standard protocol. DNA concentration and quality were estimated using a NanoDrop 2000 (ThermoFisher Scientific, Warsaw, Poland).

### 2.5 Development and analysis of DArT-seq markers

The DNA isolates were subjected for the Lupin DArTseq (1.0) procedure with 2.5 mln reads sequencing depth (Illumina Novaseq6000) by Diversity Arrays Technology Pty Ltd. (University of Canberra, Bruce, Australia). The DArTseq procedure generated two sets of markers: presence/absence (dominant) variants (named SilicoDArTs) and standard single-nucleotide polymorphisms (SNPs). SilicoDArTs may reflect several possible polymorphisms: SNPs and small indels in the recognition sites of restriction enzymes, larger insertions/deletions in the restriction fragments, as well as methylation at the restriction sites. A recently released yellow lupin C195_Genome assembly (GCA_964019355.1) was used for the DArT-seq mapping (Martinez-Hernandez et al., 2025) performed by BLAST (Altschul et al., 1990) implemented in Geneious Prime (Kearse et al., 2012) allowing 1 nt mismatch. The original DArT output was transformed into the Variant Call Format (VCF) using custom Python scripts, and the resulting VCF file was utilized in all downstream analyses. During this process, marker data were transformed into a 0, 1, 2 code, where 0 indicates the PRH444/14 allele homozygote, 2 indicates the Parys allele homozygote, and 1 indicates the heterozygote. Before linkage mapping, markers were filtered for minor allele frequency (MAF) with a threshold of 25%, missing data 34.8% and heterozygosity 5.8%. Missing data imputation was performed using the Beagle software version 4.1 (Browning and Browning, 2007) with its default settings.

### 2.6 Development and analysis of PCR markers

As in the two other Old World lupin crop species (narrow-leafed lupin and white lupin) the sequence polymorphism in regulatory regions of the *FT* homologs and some other flowering-related genes revealed an association with plant phenology and vernalization responsiveness (Nelson et al., 2017; Rychel et al., 2019; Rychel-Bielska et al., 2020, 2021, 2024; Surma et al., 2025), we decided to analyze five PCR-based markers polymorphic between the parents of RIL population (Plewiński et al., 2022). These markers, namely UNIa_M1, ELF1_F2, FTa2_F19, FTc1_F19, MFT_F1, represent the following genes: LLUT_LOCUS7777 (chromosome YL-05 at 37,301,836-37,305,897 bp), LLUT_LOCUS22604 (YL-15 at 31,942,894-31,973,052 bp), LLUT_LOCUS22662 (YL-15 at 33,730,420-33,733,344 bp), LLUT_LOCUS23834 (YL-16 at 24,624,280- 24,645,976 bp), and LLUT_LOCUS13439 (YL-09 at 10,750,526-10,753,409 bp). Detection of the FTc1_F19 marker was based on the product length difference, ELF1_F2, FTa2_F19, and MFT_F1 on were resolved by the cleaved amplified polymorphic sequence (CAPS) (Konieczny and Ausubel, 1993), while UNIa_M1 by derived CAPS (dCAPS) (Neff et al., 1998) approaches (**Supplementary Table S3**).

### 2.7 Marker segregation analysis and linkage mapping

The p-values of the Chi-square (χ2) test for Mendelian segregation in F_8_ RILs were estimated using the 1:1 expected segregation ratio (excluding heterozygotes). The calculation of probability was based on χ2 and 2 degrees of freedom. Analysis revealed a high segregation distortion in the RIL population. Mapping data was imported to Joinmap 5 (Stam, 1993) (Kyazma B.V., Wageningen, Netherlands) and the similarity of individuals was inspected. A subset of RILs revealed a high similarity to other RILs. Therefore, to reduce the number of lines with almost identical marker segregation, the RILs were distributed into 100 clusters based on genome-wide SNP variation inferred from DArTseq markers. Clustering was performed using the sparse non-negative matrix factorization (sNMF) algorithm implemented in the R package *LEA* (Frichot and François, 2015). Genotypic data were converted into the.geno format with diploid coding (0, 1, 2; missing data = 9) and subjected to sNMF analysis for K = 100–105, using 10 repetitions and 50 iterations per run. The best run was selected according to the minimum cross-entropy criterion. The resulting Q-matrix, representing the membership coefficients of individual RILs in each cluster, was used to select representative lines. For each of the 100 clusters, the RIL showing the highest membership coefficient (q-value) was chosen as the most representative genotype. This procedure resulted in a reduced set of 100 unique lines that capture the full genetic diversity of the population while minimizing redundancy. This set was supplemented with 36 RILs showing the most contrasting phenology in both directions (the earliest and the latest). For the construction of the linkage map, all markers with MAF values below 25% were removed (corresponding to the p-value of the Chi-square test of 5E-9), while the remaining markers were imported into Joinmap 5. Grouping was carried out using two methods: using the linkage LOD criterion (from 10 to 30, step 0.5) and using the recombination frequency criterion (from 0.25 to 0.01, step -0.005). Final grouping was performed at nodes common for both methods. Multipoint mapping was performed using the following parameters: mapping algorithm: maximum likelihood; spatial sampling thresholds: 0.1, 0.05, 0.03, 0.02, 0.01; map optimization rounds per sample: 5; chain length: 2500; initial acceptance probability: 0.25; cooling control parameter: 0.00025; chains without improvement limit: 50000; length of burn-in chain: 50000; Monte Carlo expectation-maximization cycles: 10; chain length per cycle: 1000; sampling period for recombination frequency matrix samples: 5. Markers with the ‘nearest neighbor fit’ and the ‘nearest neighbor stress’ values higher than 2.0 cM were removed and mapping was repeated as many times as required to maintain these values below 2.0. Markers removed during recombination frequency optimization procedure were localized in the linkage map using the Map Manager QTXb20 (Manly et al., 2001) using map function Kosambi and search linkage criterion p-value 1E-6. The same procedure was performed for selected markers with an MAF value between 20 and 25%.

### 2.8 QTL mapping

Prior to QTL mapping, heterozygote scores were replaced by homozygote scores of adjacent markers and all duplicated markers (i.e., with identical segregation) were removed, leaving a single representative for every locus. The order in linkage groups, cumulative genetic distances and LOD scores for marker pairs were exported directly from Map Manager QTXb20. The marker data set used for QTL mapping contained 136 RILs and 1448 loci distributed across 63 linkage groups with a total length of 2009.8 cM. Composite interval mapping was performed in Windows QTL Cartographer V2.5 (North Carolina State University, Raleigh, USA) using a walk speed of 1 cM, the number of background markers in the range from 5 to 25 with step 5 and window size of 3, 5, 7 cM, and 10 cM. To determine the significance threshold of the LOD score under the null hypothesis (no QTL present), a permutation test (1000×) was performed for all combinations of windows sizes and background marker numbers tested. The stability of QTLs was evaluated by comparing the results obtained for different window sizes and background marker numbers. The QTL mapping variant with a window size of 10 cM and 15 background markers was selected as the most representative. For this variant, the mapping was repeated using a walk speed of 0.5 cM. QTL regions were called for linkage group segments carrying loci with LOD values higher than the value corresponding to p=0.01 from the permutation test for a particular trait×year×photoperiod combination. The centimorgan positions of the QTL boundaries (start and end) were calculated proportionally to the decay of the trait LOD between two adjacent markers delimiting significant (LOD above p=0.01 permutation test value) and non-significant (below p=0.01) associations. QTL peaks were called at loci that showed the highest LOD values within given QTL regions. When LOD peaks for two or more trait×year×photoperiod combinations were localized within 8 cM window, they were considered to originate from the same QTL.

### 2.9 Visualization

Linkage groups were drawn in MapChart (Voorrips, 2002), sequence collinearity blocks were visualized using Circos (Krzywinski et al., 2009), and trait and marker heatmaps were drawn in Microsoft Excel.

### 2.10 Candidate genes identification

The sequences of markers localized at LOD peak loci for all QTLs were aligned by BLAST (Altschul et al., 1990) implemented in Geneious Prime (Kearse et al., 2012) to the assembly of the yellow lupin genome (Martinez-Hernandez et al., 2025) (GCA_964019355.1) and the genomic sequence for the best hit was extracted with flanking regions 250 kbp. As the annotation of the yellow lupin genome is very rudimentary, with all genes annotated as ‘putative proteins’, we transferred the annotations of the white and narrow-leafed lupin genomes. To do so, coding sequences for all yellow lupin genes from QTL regions were extracted and aligned by BLAST to white lupin and narrow-leafed lupin coding sequences deposited at NCBI as Genbank assemblies GCA_009771035.1 and GCF_001865875.1 (files GCA_009771035.1_CNRS_Lalb_1.0_cds_from_genomic.fna.gz and GCF_001865875.1_LupAngTanjil_v1.0_cds_from_genomic.fna.gz). BLAST alignment similarity threshold was set to min. 80%, coding sequence coverage to min. 50%, e-value to max 1E-50. The best hit (i.e. with the highest score) was retrieved for each gene and data for both species were combined in one list. All coding sequences annotated as ‘transcription factors’ were additionally verified using BLAST and reference RNA sequences (refseq_rna) database. Then, the putative involvement in flowering regulation of all coding sequences of yellow lupin QTL regions was evaluated by searching for the gene name in PUBMED and Google Scholar literature databases. A yellow lupin gene from the QTL region was annotated as ‘candidate gene’ if at least one orthologue in plant kingdom was reported to be involved in flowering regulation.

## 3 Results

### 3.1 The yellow lupin RIL population displayed a wide range of flowering time variation with significant correlations between years and photoperiod

**Figure 1:**
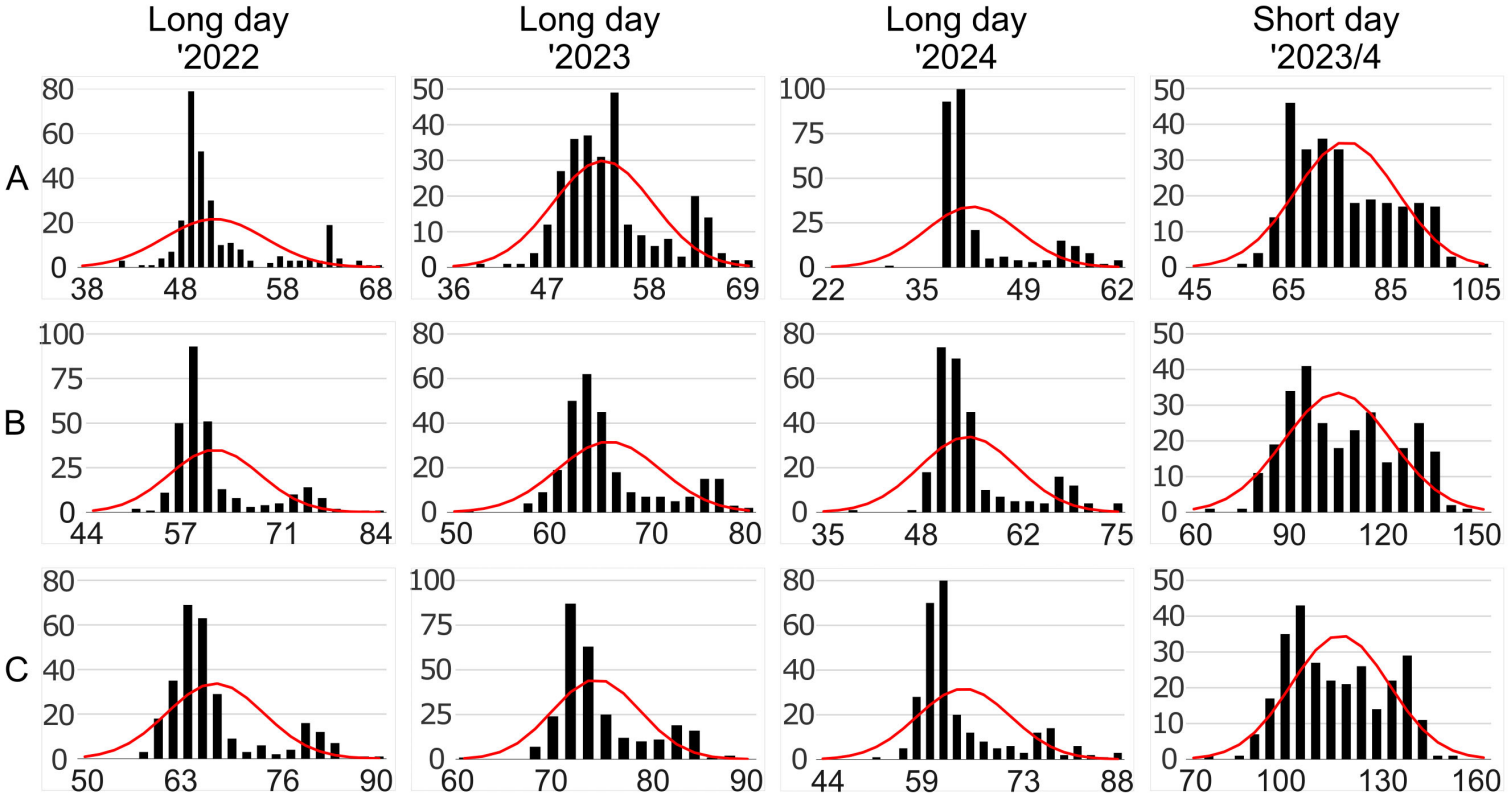
Distribution of key flowering-related traits [**(A)** floral bud emergence; **(B)** start of flowering; **(C)** end of flowering] for 276 RILs and two parental lines observed under long- and short-day photoperiods. The horizontal (x) axis describe the number of days from sowing whereas the vertical (y) axis the number of lines in a given interval. Red curves visualize normal distribution within ±3*σ* range. Observations were performed in a greenhouse at the Institute of Plant Genetics, Polish Academy of Sciences, Poznań, Poland (52°26′ N 16°54′ E) under long day (spring 2022, 2023, and 2024) and short day (winter 2023/2024) photoperiod.

Data on the number of days from sowing to floral bud emergence, start of flowering, and end of flowering were obtained for all studied genotypes (276 RILs and two parental lines, Parys and PRH444/14) during spring and summer of 2022, 2023 and 2024 years for a long-day photoperiod and during autumn and winter 2023/2024 for a short-day photoperiod (**Supplementary Tables 1**, **S2**). In the long-day photoperiod, in the first year of observations, the mean number of days from sowing to floral bud emergence ranged from 41.4 to 68.0, to the start of flowering between 48.8 and 84.0, and to the end of flowering from 57.4 to 89.6. In the second year, these values were similar, ranging from 38.8 to 67.8 days to floral bud emergence (non-significant difference between 2022 and 2023), from 51.4 to 80.2 days to the start of flowering, and from 58.0 to 86.6 days to the end of flowering. In the third year, the plant developed floral buds 30.0-64.2 days after sowing, started flowering after 38.6-77.6 days and ended flowering after 51.8-88.2 days after sowing. Under the short-day photoperiod, vegetative growth was significantly longer than under long days, since plants required 59.0 to 130.8 days to develop floral buds, 64.4 to 143.4 days to start flowering and 72.6 to 150.8 days to end of flowering. Results of inter-annual comparisons are provided in **Supplementary Table S4**. The Kolmogorov-Smirnov test revealed that all the phenotypic datasets diverged significantly from the normal distribution (**Figure 1**). Based on the observed effect size D, the magnitude of the difference between the sample and the normal distributions was lower under the short-day photoperiod than under the long-day photoperiod.

**Figure 2:**
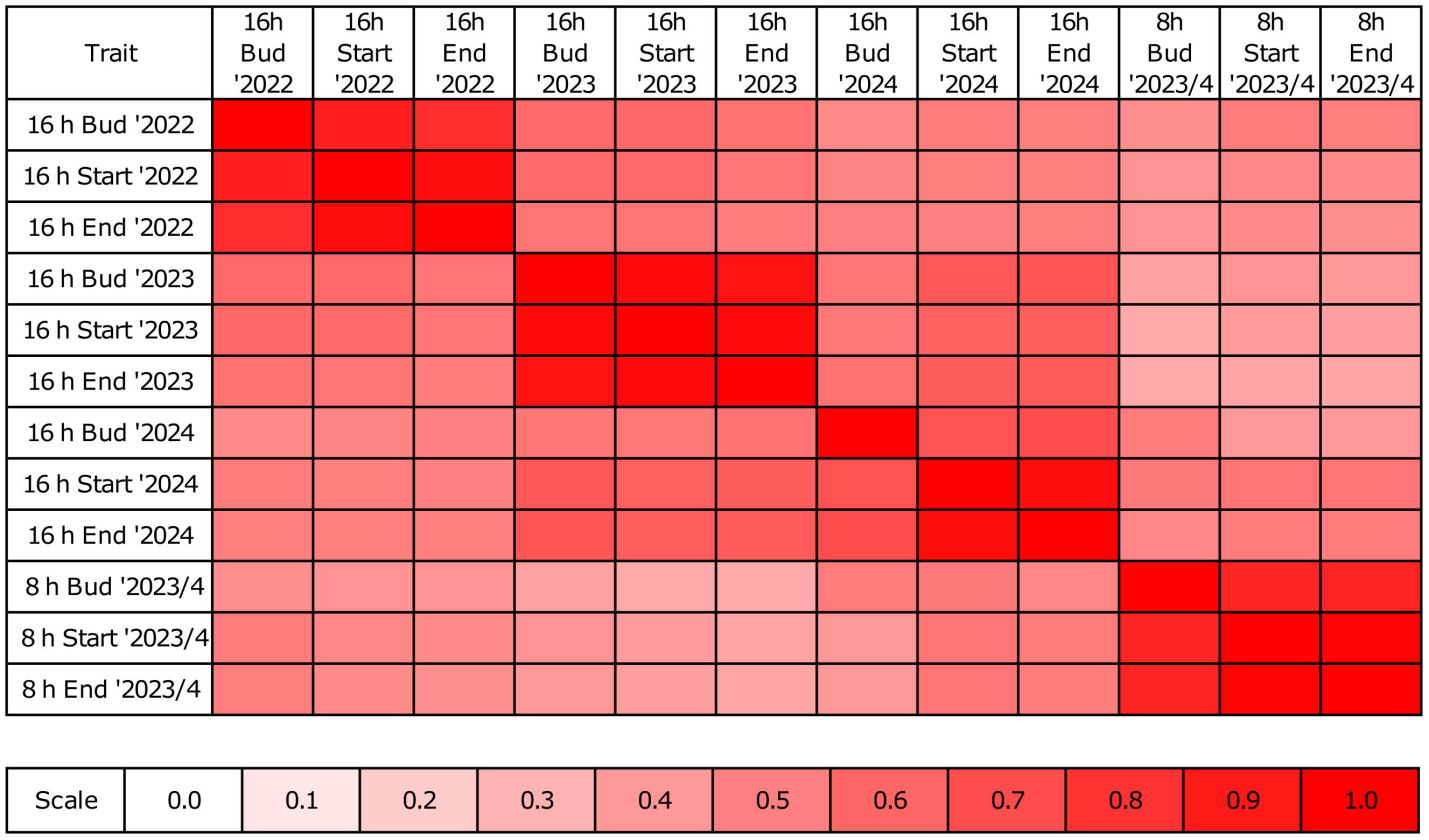
Correlation heatmap reporting Spearman rank correlation coefficients traits and years. Observations were performed in a greenhouse at the Institute of Plant Genetics, Polish Academy of Sciences, Poznań, Poland (52°26′ N 16°54′ E) under long day (spring 2022, 2023, and 2024) and short day (winter 2023/2024) photoperiod. Observed traits included the number of days from sowing to the floral bud emergence (abbreviated here as ‘Bud’), start of flowering (‘Start’) and end of flowering (‘End’). The bar below the heatmap indicates the color legend of correlation coefficients (from 0 to 1). All correlation values presented on heatmap are statistically significant (max p-value 1.2E-7).

Taking into consideration mean values from all three flowering-related traits under long-days, parental lines were positioned at the opposite edges of traits distribution, as expected for a bi-parental RIL mapping population. The Wilcoxon signed rank test (Wilcoxon, 1945) revealed that there was no significantly earlier RIL than the early parent PRH44/14, and only five RILs (P.271, P.257, P.270, P.269A, and P.186) were significantly later than the late parent Parys (**Supplementary Table S5**). These five lines reached flowering-related phenological phases 1 to 5 days later than Parys under long-day photoperiod and 7 to 17 days later under short-day photoperiod. Spearman rank correlations (Spearman, 1904) between traits within a particular year reached from 0.83 to 0.99 in 2022, from 0.93 to 0.96 in 2023, from 0.68 to 0.95 in 2024 and from 0.86 to 0.99 in the winter study of 2023/2024. The correlations between years under long-day photoperiod ranged from 0.46 to 0.67. The correlations between the photoperiods were in the range of 0.34 to 0.54 (**Figure 2**). Although the correlation coefficients span a wide range, all correlations reached statistical significance due to adequate sample size (n=278). Broad-sense heritability (**Table 1**) reached 0.87-0.88 for means, while the heritability calculated for data analyzed by linear mixed models (LMM) reached 0.97-0.98, both for the best linear unbiased estimators (BLUEs) calculated by the Cullis method (Cullis et al., 2006) and the best linear unbiased predictions (BLUPs) by the Piepho method (Piepho and Möhring, 2007).

**Table 1:** Heritability of means, genotypic and environmental variance, and genotype by environment interaction of flowering traits in yellow lupin Parys×PRH444/14 RIL population.

Trait	Mean heritability	Mean BLUPs* heritability	Mean BLUEs* heritability	Genotypic variance	Genotype by environment interaction	Environmental variance	Mean variance of a difference between BLUPs	Mean variance of a difference between BLUEs
Floral bud emergence	0.876	0.976	0.970	30.529	8.024	46.231	1.474	1.915
Start of flowering	0.871	0.978	0.977	32.798	6.865	62.852	1.436	1.532
End of flowering	0.871	0.975	0.973	34.429	10.264	50.960	1.707	1.915

*BLUEs, Best Linear Unbiased Estimators; BLUPs, Best Linear Unbiased Predictions.

### 3.2 DArT-seq genotyping provided a set of markers applicable for the construction of a linkage map anchoring all 26 yellow lupin chromosomes

The automated DNA isolation protocol yielded, on average, 16.7 ± 7.6 µg DNA per sample (min 6.3 µg, max 46.6 µg) (**Supplementary Table S6**). DArT-seq protocol provided 21216 SNP and 22478 SilicoDArT (PAV) markers. From this dataset, 92.7% of the markers had missing data below 20%, and 99.84% of the markers had a heterozygosity level not greater than 5%. However, the vast majority of the obtained DArT-seq markers were monomorphic in the given mapping population, since only 3319 of them (i.e. 7.6%) reached MAF value at least 10%, including 2302 markers (constituting 5.3% of the total number) above the 20% threshold of MAF value. After selection for missing data (max 34.8%), heterozygosity (max 5.8%) and MAF (min 12.9%), 2639 markers were retained for missing-data imputation and subsequent steps. As pairwise comparisons of RILs in JoinMap revealed significant similarities of marker segregation patterns among particular sets of RILs (exceeding 99% identity), clustering was performed to select the set of the most informative RILs, reducing the size of population used for linkage mapping to 136 lines. The grouping in the JoinMap resulted in the construction of 63 linkage groups carrying 1959 markers and some markers that were unlinked. The distribution of unlinked markers and those with a MAF value between 20 and 25% in the existing linkage groups resulted in the construction of the final genetic map that contains 2186 markers (**Supplementary Table S7**). The linkage map contains 1448 unique (non-redundant) loci that span a total genetic distance of 2009.8 cM (**Table 2**). Marker segregation is provided in **Supplementary Table S8**.

**Table 2:** Summary of markers composing the genetic linkage map constructed for the yellow lupin Parys×PRH444/14 RIL population, with their assignment to the reference genome assembly.

Linkage group	PAV markers	SNP markers	PCR markers	Unique loci	Length (cM)	Assigned pseudochromosomes	Unassigned markers
YL-01a	2	3	0	5	2.2	YL-01	0
YL-01b	14	16	0	24	33.1	YL-01	2
YL-02a	2	4	0	6	1.9	YL-02	0
YL-02b	4	8	0	12	17.8	YL-02	2
YL-02c	12	10	0	21	14.6	YL-02	4
YL-02d	14	12	0	25	13.2	YL-02	3
YL-03a	12	15	0	26	43.4	YL-03, YL-09	3
YL-03b	18	29	0	42	74.3	YL-03, YL-15	2
YL-03c	5	8	0	13	34.9	YL-03	2
YL-04a	13	19	0	27	15.0	YL-04	0
YL-04b	5	3	0	8	12.2	YL-04	2
YL-04c	10	5	0	15	16.2	YL-04	1
YL-05a	10	4	0	10	3.7	YL-05	0
YL-05b	5	2	0	7	12.2	YL-05	0
YL-05c	13	15	0	26	73.3	YL-05	3
YL-05d	52	74	1	26	19.6	YL-05, YL-07, YL-16	13
YL-06a	12	8	0	17	7.5	YL-06	2
YL-06b	2	10	0	12	18.2	YL-06, YL-02	1
YL-06c		4	0	4	1.1	YL-06	0
YL-06d	19	14	0	23	46.8	YL-06, YL-04, YL-23	3
YL-07a	5	5	0	10	19.7	YL-07	4
YL-07b	65	57	0	49	25.1	YL-07	11
YL-08	16	18	0	26	43.1	YL-08	3
YL-09	49	48	1	55	143.9	YL-09	10
YL-10	6	4	0	10	9.3	YL-10	2
YL-11	15	8	0	20	31.0	YL-11, YL-10	1
YL-12a	11	13	0	22	34.1	YL-13, YL-12	0
YL-12b	7	9	0	16	25.3	YL-12	0
YL-12c		2	0	2	0.7	YL-12	0
YL-12d	93	92	0	92	51.1	YL-12	18
YL-13a	18	16	0	23	26.1	YL-13	5
YL-13b	22	13	0	24	20.8	YL-13	6
YL-13c	9	10	0	18	14.1	YL-13	0
YL-14a	3	6	0	8	2.6	YL-14	0
YL-14b	2	2	0	4	5.4	YL-14	0
YL-14c	101	93	0	82	79.2	YL-14	18
YL-14d	9	4	0	10	24.0	YL-14, YL-11	0
YL-15a	29	27	0	40	51.4	YL-15	13
YL-15b	17	25	0	14	30.6	YL-09, YL-23, YL-15	2
YL-15c		3	0	3	1.5	YL-15	0
YL-16a	28	31	1	51	61.2	YL-16	5
YL-16b	7	6	0	12	11.0	YL-16	4
YL-17	33	28	0	55	152.2	YL-17, YL-18	8
YL-18a	11	8	1	11	28.4	YL-15, YL-18, YL-05	1
YL-18b	4	8	0	12	28.1	YL-18	1
YL-18c	7	11	0	13	71.3	YL-18	0
YL-19a	11	10	0	10	3.3	YL-19	0
YL-19b	2	3	0	5	6.5	YL-19	2
YL-20a	6	8	0	14	47.6	YL-20	0
YL-20b	17	22	0	37	31.4	YL-20	0
YL-21a	41	45	0	56	61.1	YL-21, YL-07	8
YL-21b	3	5	0	6	8.7	YL-21	0
YL-22a	8	4	0	12	26.8	YL-22	1
YL-22b	17	13	0	29	29.2	YL-22	3
YL-23a	59	60	0	67	89.8	YL-23, YL-10	18
YL-23b	34	38	0	53	78.3	YL-23, YL-04	9
YL-24	22	23	0	33	59.2	YL-24	1
YL-25a	9	13	0	17	7.5	YL-25	1
YL-25b	2	4	0	6	5.0	YL-25	0
YL-25c	2	0	0	2	0.7	YL-25	0
YL-26a	31	17	0	37	67.2	YL-26, YL-02, YL-18	6
YL-26b	24	26	0	31	34.5	YL-15, YL-26, YL-07, YL-25, YL-03	8
YL-26c	2	0	0	2	0.4	YL-26	0

**Figure 3:**
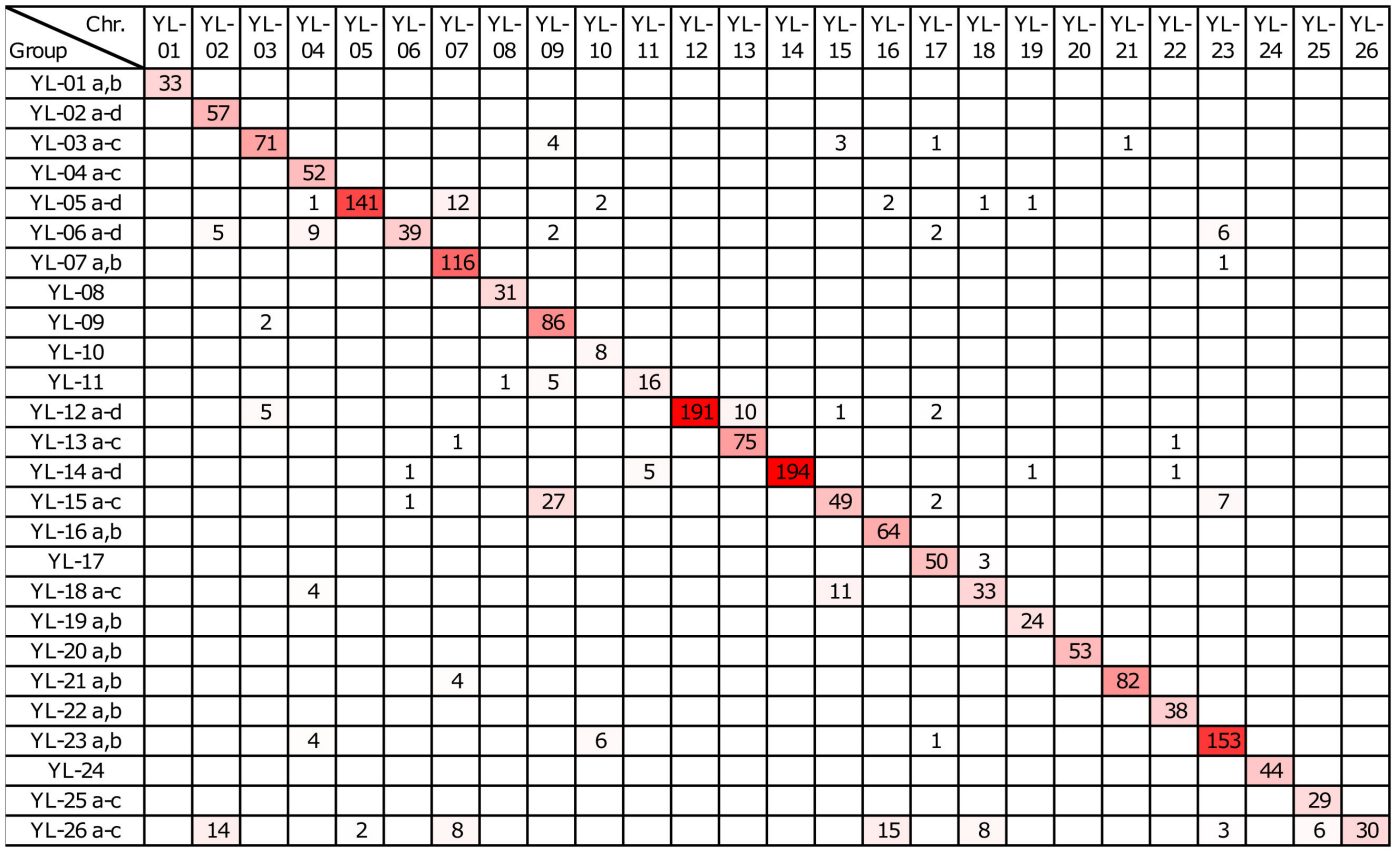
Assignment of linkage groups to pseudochromosomes of the yellow lupin genome. Heatmap shows the number of markers matching a particular pseudochromosome in the scale from white (0) to red (maximum, 194). Sequences of 2186 markers were assigned to the genome assembly GCA_964019355.1.

Based on the marker sequence alignments, 1974 markers were mapped to the yellow lupin genome (GCA_964019355.1) matching the 26 yellow lupin chromosomes, while 212 (9.7%) remained unassigned (**Supplementary Table S7**). A high consistency between the genetic linkage of particular markers and their physical linkage in the pseudochromosomes was revealed (**Figure 3**); therefore, linkage groups were renamed to follow the names used in the current assembly of the yellow lupin genome (i.e. groups YL-01a and YL-01b correspond to the chromosome YL-01, groups YL-02a, YL-02b, YL-02c and YL-02d to the chromosome YL-02, etc.). The largest linkage groups are YL-14c, YL-12d, YL-05d, YL-07b and YL-23a, carrying 194, 185, 127, 122, and 119 markers, respectively. The smallest linkage groups are YL-01a, YL-19b, YL-06c, YL-14b, YL-15c, YL-12c, YL-25c, and YL-26c, carrying 2 to 5 markers. All linkage groups containing at least five markers are composed of both types of DArT-seq markers (SNPs and PAVs), indicating their relatively uniform distribution in mapped regions of the genome. PCR-based markers anchored in flowering pathway-related genes were mapped in four linkage groups, namely YL-05d (UNIa_M1), YL-09 (MFT_F1), YL-16a (FTc1_F19) and YL-18a (ELF1_F2).

In total, 290 markers present in the current version of the linkage map showed significant segregation distortion from the expected 1: 1 pattern below the p-value threshold of <1E-7, including 132 heavily distorted markers (p-value <1E-10). Based on the p-value threshold of the latter Chi-square test, the highest numbers of distorted markers were observed in the linkage groups YL-12a, YL-23b, YL-26a, YL-15b, YL-18a, YL-06d, and YL-26b, each carrying 11 to 23 such distorted markers. As many as 49 linkage groups revealed no distorted markers at this p-value threshold, while 41 linkage groups also when used a more relaxed criterion of 1E-7. The largest, in the context of genetic distance, blocks of distorted markers were localized in linkage groups YL-26a (33.6 cM, 27 markers), YL-06d (31.3 cM, 24 markers), YL-15b (30.6 cM, 42 markers), YL-17 (26.6 cM, 20 markers), YL-12a (26.5 cM, 18 markers), YL-11 (19.1 cM, 6 markers), and YL-26b (13.1 cM, 22 markers).

Comparative mapping of the linkage groups and pseudochromosomes revealed that 89.1% of markers assigned to pseudochromosomes were mapped in syntenic linkage groups (**Figure 3**). Indeed, all markers of six chromosomes (YL-01, YL-12, YL-14, YL-20, YL-24, and YL-26) were mapped only in the syntenic linkage groups, whereas other six chromosomes (YL-08, YL-21, YL-05, YL-06, YL-19, and YL-22) revealed no more than 1–2 markers mapped in non-syntenic linkage groups. On the other hand, eight chromosomes (YL-18, YL-15, YL-16, YL-23, YL-04, YL-02, YL-07, YL-09) revealed more than 10 markers dispersed among non-syntenic linkage groups. It should be noted that misaligned markers were usually also heavily distorted, therefore, observed inconsistencies between genetic and genomic marker positions do not undermine the quality of genome assembly.

### 3.3 QTL mapping revealed one major locus significant for all experimental variants and thirteen minor loci significant for at least two trait×year×photoperiod combinations

**Figure 4:**
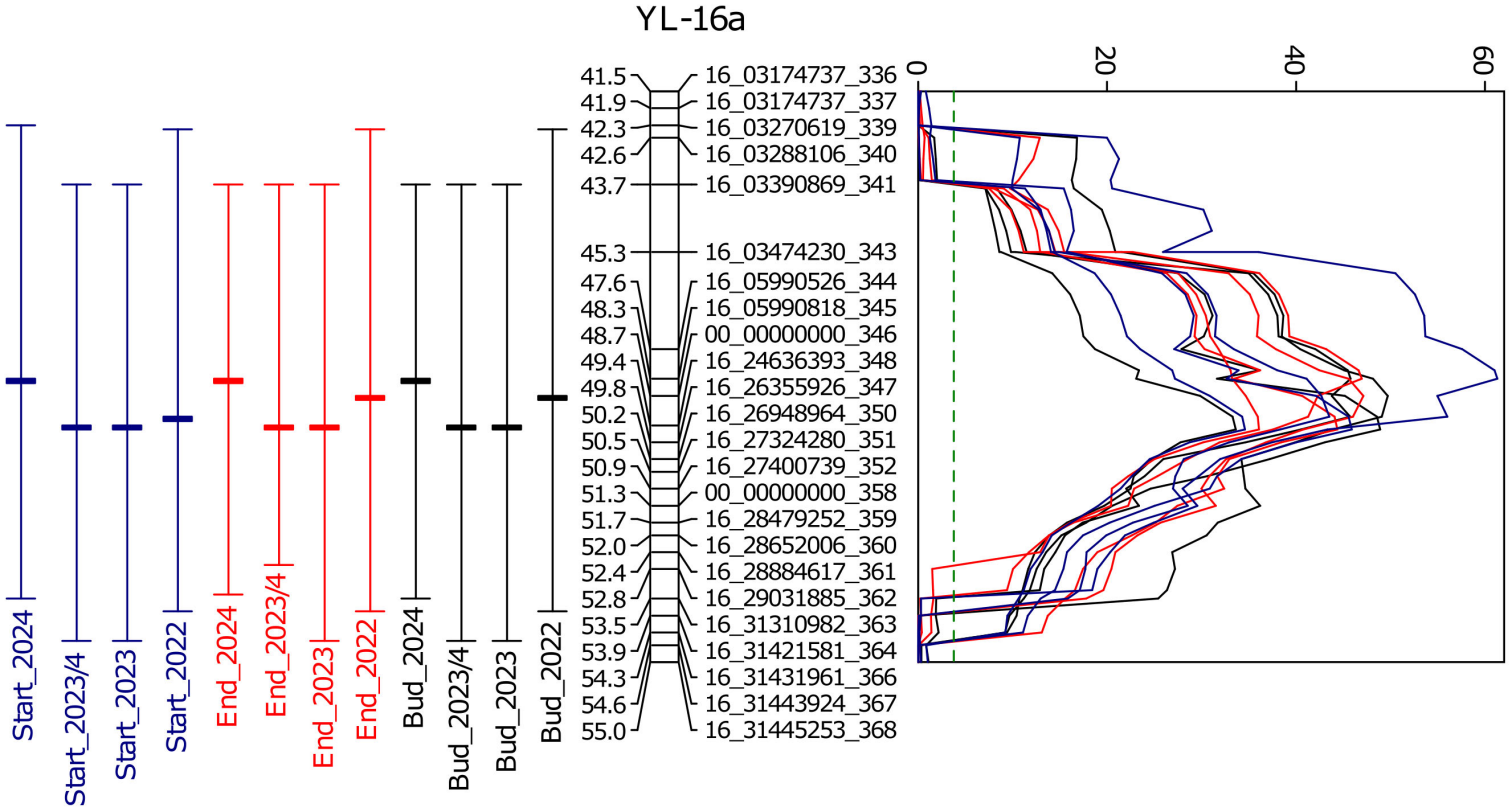
Segment of the linkage group YL-16a from the yellow lupin linkage map carrying newly developed DArT-seq markers and the major QTL, quantitative trait locus for flowering phenology traits identified in the Parys×PRH444/14 recombinant inbred line mapping population. Composite interval mapping was performed for 136 RILs and 1448 non-redundant marker loci using 10 background markers and 15 cM window size. Bars located on the left side of the linkage group indicate QTL boundaries and LOD peak positions, whereas a graph located on the right side shows LOD values for particular traits. LOD threshold (P = 0.01) was determined by permutation test (n=1000). Traits are expressed as the number of days from sowing to the floral bud emergence (abbreviated here as ‘Bud’), start of flowering (‘Start’), and end of flowering (‘End’). Observations were performed in a greenhouse at the Institute of Plant Genetics, Polish Academy of Sciences, Poznań, Poland (52°26′ N 16°54′ E) under long day (spring 2022, 2023, and 2024) and short day (winter 2023/2024) photoperiod.

QTL mapping was carried out for three traits, measured as the number of days from sowing to reaching particular developmental phases (floral bud emergence, start of flowering, and end of flowering), and for four year×photoperiod combinations (short-day photoperiod in winter season 2023/2024 and long-day photoperiod in spring/summer seasons 2022, 2023 and 2024), therefore 12 trait×year×photoperiod combinations were analyzed in total. The initial mapping performed with 20 combinations of window size (3–10 cM) and background marker number (5-25) revealed the presence of numerous QTLs distributed across 40 linkage groups (**Supplementary Table S9**), with the most represented linkage group YL-16a (significant for all 240 combinations) and YL-14c (significant for 83 combinations). The QTL mapping for, selected as representative, window size 10 cM and background number 15, revealed 33 statistically significant QTL regions (**Supplementary Table S10**). These regions included one major QTL, localized in a linkage group YL-16a, significant for all 12 combinations and manifesting high LOD peaks characterized by maximum values between 33.6 and 61.3 (**Table 3**; **Figure 4**). Phenotypic variance explained by this QTL ranged from 16 to 33.9%, while the additive effect ranged from 4.77 to 17.74 days. The marker-trait correlation coefficients for this QTL were very high and ranged from 0.73 to 0.92 for the LOD peak loci (**Supplementary Table S11**). The directions of additive effects were consistent with the marker-trait correlations for all associations in this QTL. It is an expected outcome for highly correlated markers, because the additive effect estimates the change in phenotype upon replacement of the QTL allele from one parent by the other parent, while the markers were encoded according to the scores of the parental lines (early parent 0, late parent as 2).

**Table 3:** The major QTL for phenology traits (significant for all trait×year ×photoperiod combinations) identified in the yellow lupin Parys×PRH444/14 RIL population.

Linkage group	Trait	Year	Photoperiod	QTL start cM	QTL peak cM	QTL end cM	QTL peak LOD	Explained variance (%)	Marker-trait correlation	Additive effect
YL-16a	BE*	2022	Long	42.35	48.70	53.84	49.71	33.74	0.89	5.43
YL-16a	BE	2023	Long	43.69	49.45	54.48	48.90	22.12	0.87	6.11
YL-16a	BE	2023/4	Short	43.70	49.45	54.48	33.61	28.16	0.76	16.40
YL-16a	BE	2024	Long	43.69	48.33	53.47	45.77	16.76	0.92	8.13
YL-16a	SF*	2022	Long	42.41	49.20	53.80	43.51	23.91	0.89	6.46
YL-16a	SF	2023	Long	43.65	49.45	54.50	45.88	16.01	0.88	4.80
YL-16a	SF	2023/4	Short	43.68	49.45	54.49	34.60	25.52	0.73	15.07
YL-16a	SF	2024	Long	42.33	48.33	53.48	61.31	28.03	0.89	6.93
YL-16a	EF*	2022	Long	42.39	48.70	53.81	47.12	24.79	0.90	6.69
YL-16a	EF	2023	Long	43.69	49.45	54.53	44.38	18.36	0.88	4.77
YL-16a	EF	2023/4	Short	43.70	49.45	52.68	36.05	33.91	0.73	17.74
YL-16a	EF	2024	Long	43.68	48.33	53.43	46.93	23.53	0.89	8.96

*traits: BE, floral bud emergence; SF, start of flowering; EF, end of flowering.

**Figure 5:**
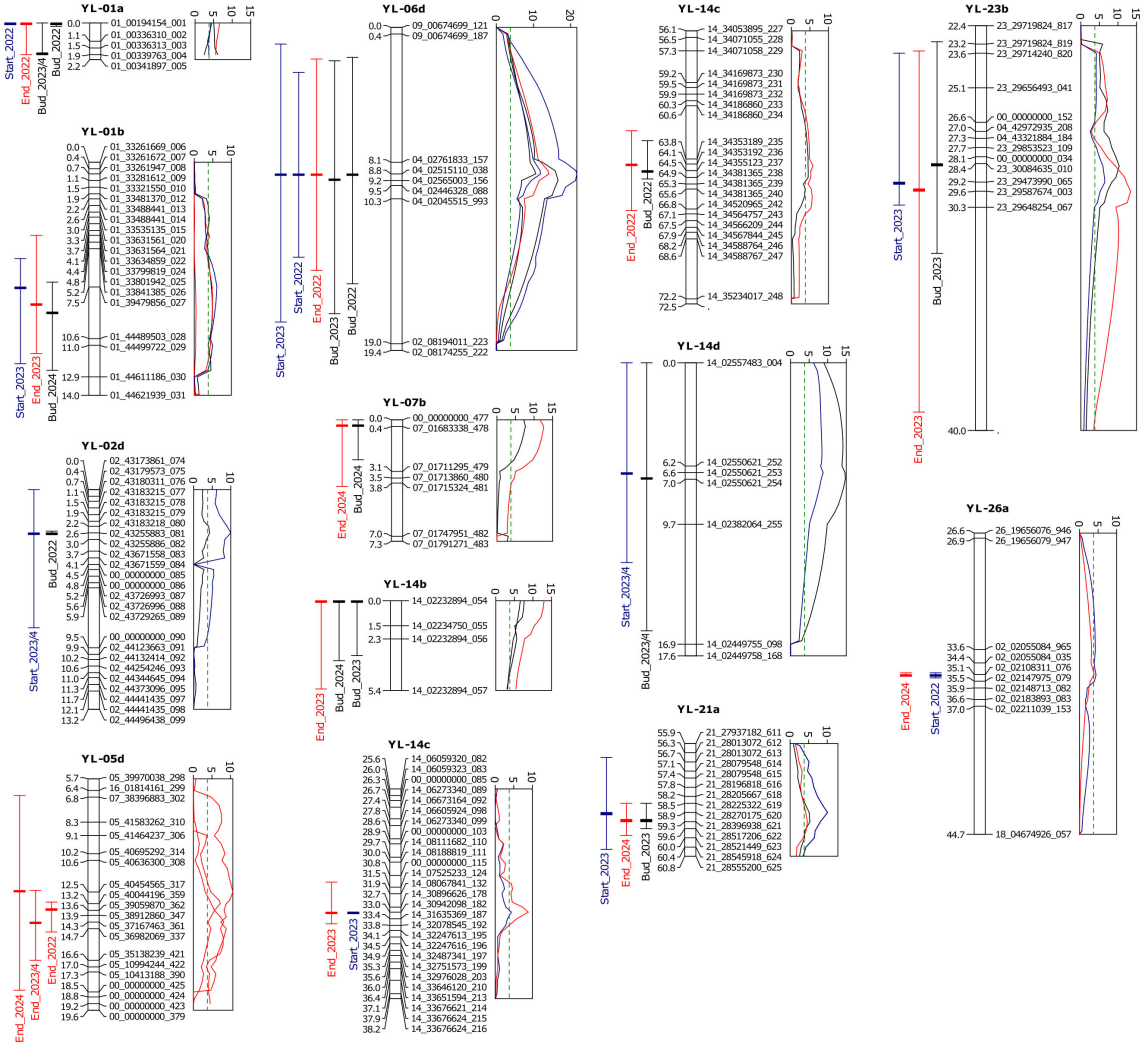
Segment of the linkage groups from the yellow lupin linkage map carrying newly developed DArT-seq markers and minor QTLs, quantitative trait loci for flowering phenology traits identified in the Parys×PRH444/14 recombinant inbred line mapping population. Composite interval mapping was performed for 136 RILs and 1448 non-redundant marker loci using 10 background markers and 15 cM window size. Bars located on the left side of the linkage group indicate QTL boundaries and LOD peak positions, whereas a graph located on the right side shows LOD values for particular traits. LOD threshold (P = 0.01) was determined by permutation test (n=1000). Traits are expressed as the number of days from sowing to the floral bud emergence (abbreviated here as ‘Bud’), start of flowering (‘Start’), and end of flowering (‘End’). Observations were performed in a greenhouse at the Institute of Plant Genetics, Polish Academy of Sciences, Poznań, Poland (52°26′ N 16°54′ E) under long day (spring 2022, 2023, and 2024) and short day (winter 2023/2024) photoperiod.

Apart from this major YL-16a locus, 13 were identified QTLs significant for at least two trait× year×photoperiod combinations, named ‘minor QTLs’, in 12 linkage groups: YL-01a, YL-01b, YL-02d, YL-05d, YL-06d, YL-07b, YL-14b, YL-14c, YL-14d, YL-21a, YL-23b, and YL-26a (**Table 4**; **Figure 5**). Ten minor QTLs revealed significant associations for at least two traits from the same year, which is logical consequence of their partial dependence (i.e., late floral bud emergence implicates late start of flowering and late end of flowering of the same plant in the same experiment). On the other hand, a QTL mapped in YL-05d was significant only for the end of flowering, but in both photoperiods. Seven minor QTLs showed LOD peak values above 10, with the highest values reported for YL-06d (max. 21.7) and YL-14d (max. 14.8). Interestingly, the latter QTL was specific for the short-day photoperiod, whereas the preceding one was specific for the long day. The phenotypic variance explained by all minor QTLs ranged from 0.7 to 8.6%, with values above 4% estimated for QTLs mapped in YL-02d, YL-05d, YL-06d, YL-14d. The additive effects for minor QTLs ranged from -8.63 to 7.44 days, while the marker-trait correlations ranged from -0.57 to 0.70. Moreover, for all minor QTLs, the sign of marker-trait correlation coefficients was coherent within a QTL, however, in two QTLs, located in YL-02d and YL-05d, directions of additive effects varied between traits or years. Seven minor QTLs revealed the same directions of additive effects and marker-trait correlations, including three QTLs with negative additive effects (YL-06d, YL-14b, and YL-14c). These three QTLs are potential candidates for a possible further shortening of the vegetative phase in yellow lupin by the introduction of opposite alleles into the rapid flowering PRH444/14 line.

**Table 4:** Minor QTLs for flowering phenology traits (significant for at least two trait×year×photoperiod combinations) identified in the yellow lupin Parys×PRH444/14 RIL population.

Linkage group	Trait	Year	Photoperiod	QTL start cM	QTL peak cM	QTL end cM	QTL peak LOD	Explained variance (%)	Marker-trait correlation	Additive effect
YL-01a	BE*	2022	Long	0.01	0.01	0.16	4.26	1.20	0.37	-1.04
YL-01a	BE	2023/4	Short	0.01	1.88	1.92	6.11	3.10	0.20	-4.65
YL-01a	SF*	2022	Long	0.01	0.01	0.14	4.43	1.18	0.38	-1.18
YL-01a	EF*	2022	Long	0.01	0.01	1.90	6.59	1.53	0.37	-1.43
YL-01b	BE	2024	Long	7.21	9.00	12.50	4.89	0.70	0.55	1.04
YL-01b	SF	2023	Long	5.75	7.50	12.09	6.11	1.00	0.64	0.96
YL-01b	EF	2023	Long	4.35	8.50	11.54	4.91	1.00	0.64	0.88
YL-02d	SF	2023/4	Short	0.01	2.60	8.29	10.01	4.63	-0.09	-6.58
YL-02d	BE	2022	Long	2.45	2.60	2.66	4.32	1.22	-0.42	1.30
YL-05d	EF	2024	Long	6.72	12.47	18.38	10.61	2.22	0.66	-3.00
YL-05d	EF	2022	Long	13.06	13.59	14.87	5.66	1.33	0.70	2.07
YL-05d	EF	2023/4	Short	12.41	14.33	16.57	8.16	4.23	0.42	-8.63
YL-06d	BE	2022	Long	1.79	8.81	15.43	10.57	3.64	-0.30	-2.26
YL-06d	BE	2023	Long	2.03	9.18	17.18	16.61	3.88	-0.32	-2.46
YL-06d	SF	2023	Long	0.95	8.81	17.71	21.67	4.65	-0.34	-2.69
YL-06d	SF	2022	Long	2.73	8.81	13.81	11.88	3.61	-0.30	-2.86
YL-06d	EF	2022	Long	1.92	8.81	14.58	14.13	3.88	-0.30	-3.05
YL-07b	EF	2024	Long	0.01	0.38	3.96	12.60	2.81	0.61	2.78
YL-07b	BE	2024	Long	0.01	0.38	2.41	7.67	1.41	0.61	1.60
YL-14b	BE	2023	Long	0.01	0.01	3.34	6.59	1.31	-0.27	-1.27
YL-14b	BE	2024	Long	0.01	0.01	3.58	7.62	1.08	-0.30	-1.32
YL-14b	EF	2023	Long	0.01	0.01	5.27	13.00	2.90	-0.33	-1.64
YL-14c	SF	2023	Long	33.00	33.04	33.10	4.34	0.68	0.28	-1.45
YL-14c	EF	2023	Long	31.24	33.04	33.73	8.90	1.84	0.29	-2.62
YL-14c	BE	2022	Long	62.69	64.53	65.00	4.54	1.28	-0.57	-1.11
YL-14c	EF	2022	Long	62.05	64.16	66.93	5.84	1.38	-0.57	-1.45
YL-14d	BE	2023/4	Short	0.01	6.98	16.07	14.75	8.61	0.49	7.44
YL-14d	SF	2023/4	Short	0.01	6.61	12.03	8.75	4.25	0.49	5.98
YL-21a	BE	2023	Long	58.48	59.29	59.63	5.34	1.04	-0.34	1.25
YL-21a	SF	2023	Long	43.83	49.86	52.56	12.86	2.35	-0.37	-2.39
YL-21a	EF	2024	Long	58.53	59.29	59.90	4.65	0.87	-0.38	1.33
YL-23b	BE	2023	Long	23.13	28.46	32.28	10.32	2.19	0.45	2.07
YL-23b	SF	2023	Long	23.57	29.20	30.16	6.47	1.05	0.42	1.25
YL-23b	EF	2023	Long	23.48	29.57	39.20	13.38	3.20	0.39	2.05
YL-26a	SF	2022	Long	34.99	35.14	35.25	4.45	1.17	-0.19	1.62
YL-26a	EF	2024	Long	34.97	35.14	35.19	3.92	0.73	-0.05	1.52

*traits: BE, floral bud emergence; SF, start of flowering; EF, end of flowering.

In addition to one major QTL and 13 minor ones, we identified 19 QTLs, named here as unique QTLs, which were significant only for single trait×year×photoperiod combinations (**Table 5**). All these QTLs revealed LOD peak values below 10, with a maximum value of 8.1 reported for YL-07a. Phenotypic variance explained by unique QTLs ranged from 0.7 to 3.9%, whereas additive effects from -3.85 to 7.03 days. The correlations ranged from -0.45 to 0.64. As these QTLs represent single associations, the possibilities of controlling false positives are limited. Some support may be provided by genetic linkage for QTL pairs localized in the same linkage groups, such as YL-03b (8.8 cM of genetic distance between loci), YL-17 (30.5 cM) and YL-09 (47.3 cM). Nevertheless, only a pair of QTLs located in YL-17 revealed the same direction of correlations and additive effects. Moreover, a unique QTL in YL-07b was localized only 13.6 cM away from a minor QTL significant for two trait×year×photoperiod combinations, having the same direction of correlation and additive effects.

**Table 5:** Unique QTLs for flowering phenology traits (significant for one trait×year×photoperiod combination) identified in yellow lupin Parys×PRH444/14 RIL population.

Linkage group	Trait	Year	Photoperiod	QTL start cM	QTL peak cM	QTL end cM	QTL peak LOD	Explained variance (%)	Marker-trait correlation	Additive effect
YL-02c	SF*	2023	Long	6.27	8.14	8.66	4.35	0.76	0.17	-0.76
YL-03b	EF*	2022	Long	61.44	65.32	69.30	7.30	2.11	-0.25	-2.12
YL-03b	EF	2023/4	Short	72.64	74.09	74.09	7.68	3.59	0.01	6.59
YL-05c	SF	2023/4	Short	62.33	66.47	69.22	4.75	2.95	0.07	-3.85
YL-07a	BE*	2023	Long	0.01	0.76	7.38	8.07	1.64	-0.19	-1.40
YL-07b	SF	2022	Long	13.93	14.00	14.17	4.62	1.37	0.64	1.91
YL-09	EF	2022	Long	93.42	96.33	97.54	6.68	1.78	0.09	-1.46
YL-09	SF	2023	Long	131.40	143.65	143.65	7.57	1.30	0.41	1.10
YL-13a	EF	2023	Long	7.53	10.05	12.42	4.35	2.30	0.64	-1.60
YL-13c	EF	2024	Long	0.01	0.38	1.39	6.59	1.28	-0.36	2.42
YL-13c	SF	2024	Long	6.02	11.85	13.71	7.39	1.09	-0.41	-1.26
YL-15b	BE	2024	Long	13.73	16.63	16.99	5.62	0.73	-0.45	1.09
YL-16b	EF	2022	Long	0.01	0.01	0.41	5.00	1.15	-0.38	-1.64
YL-17	BE	2023	Long	91.75	91.81	92.10	4.12	0.81	0.28	-1.14
YL-17	EF	2024	Long	121.98	122.34	122.63	4.12	0.77	0.58	-1.28
YL-20a	EF	2023/4	Short	0.01	0.01	5.58	7.53	3.43	0.59	5.98
YL-20b	EF	2024	Long	17.66	22.39	27.27	4.68	0.88	-0.29	-1.41
YL-23a	BE	2023/4	Short	15.40	17.67	21.97	6.64	3.91	0.53	7.03
YL-24	SF	2023/4	Short	55.39	57.27	58.86	7.21	3.69	-0.19	6.65

*traits: BE, floral bud emergence; SF, start of flowering; EF, end of flowering.

### 3.4 The majority of QTLs are located in regions carrying known regulators of flowering time

**Figure 6:**
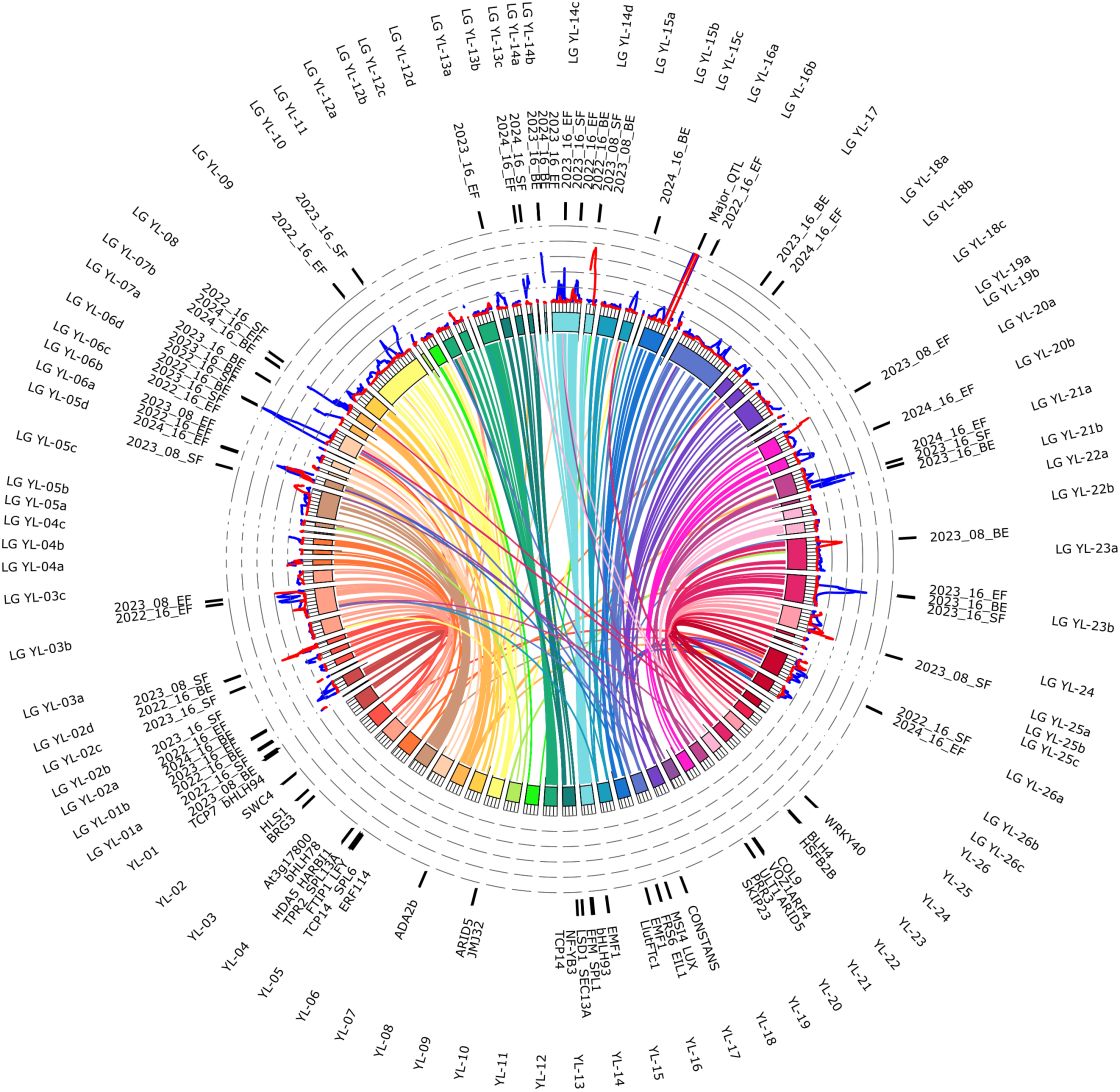
Collinearity links matching yellow lupin linkage groups (YL-01a – YL-026c) and pseudochromosomes (YL-01 – YL-26). Ribbons symbolize homologous links identified by DNA sequence similarity. Chromosomes and linkage groups are drawn to scale 1 cM = 1 Mbp. The tick values provide genetic (linkage groups, 10 cM) and physical (chromosomes, 10 Mb) distances. Labels localized outside the ideogram circle indicate QTLs (on linkage groups) and flowering time pathway regulatory genes (on chromosomes). Traits are expressed as the number of days from sowing to the floral bud emergence (abbreviated here as ‘Bud’), start of flowering (‘Start’), and end of flowering (‘End’). Observations were performed in a greenhouse at the Institute of Plant Genetics, Polish Academy of Sciences, Poznań, Poland (52°26′ N 16°54′ E) under long day (spring 2022, 2023, and 2024) and short day (winter 2023/2024) photoperiod. Circular graphs show maximum LOD values calculated for time to flowering: blue (long days) and red (short days) in a scale from 0 to 20.

To investigate whether identified QTLs are located in genome regions that carry potential genes from flowering regulatory pathways, we annotated genomic regions flanking all identified QTLs 250 kbp in both directions (500 kbp in total) and searched literature databases for publication records about identified genes. For some QTLs (YL-01b, YL-05c, YL-05d and YL-16b) analyzed genome segments were larger than 500 kbp due to redundant markers or a gap between markers flanking LOD peaks. We designated candidate genes (**Figure 6**) for 26 QTLs, including 11 minor QTLs (**Table 6**) and 14 unique QTLs (**Table 7**). For the major QTL in YL-16a, only one gene, *LlutFTc1*, was selected as potentially associated with flowering regulation. For the minor QTL in YL-05d, we selected 6 genes: *ETHYLENE RESPONSE FACTOR114* (*ERF114*), *SQUAMOSA PROMOTER BINDING PROTEIN-LIKE13A* (SPL13A), *HARBINGER TRANSPOSASE DERIVED1* (*HARBI1*), *FT-INTERACTING PROTEIN1* (*FTIP1*) and *TOPLESS-RELATED2* (*TPR2*). Two genes were selected for each of the following minor QTLs: YL-01b, *BASIC HELIX–LOOP–HELIX94* (*bHLH94*) and *TEOSINTE BRANCHED1*, *CYCLOIDEA, PROLIFERATING CELL FACTOR7* (*TCP7*); YL-06d, *At3g17800* and *bHLH78*; YL-14c, *LYSINE SPECIFIC DEMETHYLASE1* (LSD1) and *SQUAMOSA PROMOTER BINDING PROTEIN-LIKE1* (*SPL1*); and YL-21a, *AUXIN RESPONSE FACTOR4* (*ARF4*) and *PSEUDORESPONSE REGULATOR3* (*PRR3*). Single candidate genes were nominated for the following minor QTLs: YL-02d, *SWR1 COMPLEX4* (*SWC4*); YL-07b, *UV-B-INDUCED PROTEIN TRANSCRIPTIONAL ADAPTER2b* (*ADA2b*); YL-14b, *EMBRYONIC FLOWER1* (*EMF1*); YL-14c, *EARLY FLOWERING MYB* (*EFM*); YL-14d, *bHLH93*; YL-23b, *HEAT SHOCK TRANSCRIPTION FACTOR B2B* (*HSFB2B*) (**Table 6**).

**Table 6:** Candidate genes from flowering regulation pathways selected for major and minor QTLs identified in yellow lupin Parys×PRH444/14 RIL population for flowering phenology traits.

Linkage group	Flanking marker or GenBank protein	Flanking marker position on map	Gene name	Chromosome name	Chromosome min. position	Reference
YL-01b	PAV18953	7.49 cM	–	YL-01	39 479 856	–
YL-01b	CAL0300077.1	–	bHLH94-like	YL-01	39 646 670	(Lin et al., 2019)
YL-01b	CAL0300503.1	–	TCP7-like	YL-01	44 331 462	(Li et al., 2021; Tian et al., 2025)
YL-01b	PAV18138	10.62 cM	–	YL-01	44 489 503	–
YL-02d	CAL0302374.1	–	SWC4-like	YL-02	43 120 000	(Gómez-Zambrano et al., 2018)
YL-02d	SNP03493	0.00 cM	–	YL-02	43 173 861	–
YL-02d	PAV18528	2.59 cM	–	YL-02	43 255 883	–
YL-05d	SNP06639	14.32 cM	–	YL-05	37 167 463	–
YL-05d	CAL0306717.1	–	LFY-like	YL-05	37 301 836	(Parcy et al., 1998; Wagner et al., 1999)
YL-05d	CAL0306718.1	–	ERF114-like	YL-05	37 329 212	(Fei et al., 2025)
YL-05d	CAL0306747.1	–	SPL13A-like	YL-05	38 070 203	(Yamagishi et al., 2024; Ye et al., 2024)
YL-05d	CAL0306775.1	–	HARBI1-like	YL-05	38 572 255	(Zhou et al., 2021; Zhao et al., 2026)
YL-05d	CAL0306806.1	–	FTIP1-like	YL-05	39 228 346	(Liu et al., 2012; Fang et al., 2024)
YL-05d	CAL0306858.1	–	TPR2-like	YL-05	39 832 300	(Chang et al., 2019; Plant et al., 2021)
YL-05d	SNP02307	12.46 cM	–	YL-05	40 310 407	–
YL-06d	SNP17296	10.29 cM	–	YL-04	2 045 515	–
YL-06d	CAL0304548.1	–	At3g17800-like	YL-04	2 114 386	(Liu et al., 2022)
YL-06d	CAL0304552.1	–	bHLH78-like	YL-04	2 154 232	(Liu et al., 2013; Yao et al., 2025)
YL-06d	SNP11841	8.80 cM	–	YL-04	2 515 110	–
YL-07b	PAV19453	0.37 cM	–	YL-07	1 683 338	–
YL-07b	CAL0309128.1	–	ADA2b-like	YL-07	1 858 699	(Wu et al., 2021)
YL-07b	PAV18171	8.81 cM	–	YL-07	2 160 927	–
YL-14b	CAL0319235.1	–	EMF1-like	YL-14	2 206 454	(Park et al., 2011; Pu et al., 2013)
YL-14b	PAV18716	0.00 cM	–	YL-14	2 232 894	–
YL-14c	SNP01887	33.03 cM	–	YL-14	31 024 966	–
YL-14c	CAL0320068.1	–	EFM-like	YL-14	31 412 372	(Yan et al., 2014)
YL-14c	SNP14255	33.03 cM	–	YL-14	31 456 809	–
YL-14c	SNP09704	60.65 cM	–	YL-14	34 186 860	–
YL-14c	CAL0320196.1	–	LSD1-like	YL-14	34 201 428	(Jiang et al., 2007)
YL-14c	CAL0320206.1	–	SPL1-like	YL-14	34 364 680	(Preston and Hileman, 2010; Jung et al., 2012)
YL-14c	PAV18468	64.89 cM	–	YL-14	34 381 365	–
YL-14d	PAV18737	6.60 cM	–	YL-14	2 550 621	–
YL-14d	PAV18863	0.00 cM	–	YL-14	2 557 483	–
YL-14d	CAL0319281.1	–	bHLH93-like	YL-14	2 775 075	(Sharma et al., 2016; Poirier et al., 2018)
YL-16a	FTc1_F19	49.44 cM	–	YL-16	24 636 393	–
YL-16a	CAL0322774.1	–	LlutFTc1	YL-16	24 639 654	(Nelson et al., 2017; Plewiński et al., 2022)
YL-16a	PAV20130	49.81 cM	–	YL-16	26 355 926	–
YL-21a	SNP18666	59.28 cM	–	YL-21	28 396 938	–
YL-21a	CAL0329092.1	–	ARF4-like	YL-21	28 406 952	(Dong et al., 2021, 2022)
YL-21a	CAL0329111.1	–	PRR3-like	YL-21	28 550 453	(Murakami et al., 2004; Li et al., 2020)
YL-21a	SNP18837	60.76 cM	–	YL-21	28 555 200	–
YL-23b	PAV19537	29.19 cM	–	YL-23	29 473 990	–
YL-23b	CAL0331976.1	–	HSFB2B-like	YL-23	29 229 574	(Jeong et al., 2022)

**Table 7:** Candidate genes from flowering regulation pathways selected for unique QTLs.

Linkage group	Flanking marker or GenBank protein	Flanking marker position on map	Gene name	Chromosome name	Chromosome min. position	Reference
YL-02c	SNP00964	4.1 cM	–	YL-02	1 485 679	–
YL-02c	CAL0300967.1	–	HLS1-like	YL-02	1 507 668	(Wang et al., 2023; Nobusawa et al., 2025)
YL-02c	PAV18507	11.6 cM	–	YL-02	1 513 723	–
YL-03b	PAV18091	71.3 cM	–	YL-03	43 898 105	–
YL-03b	CAL0304275.1	–	BRG3-like	YL-03	44 332 079	(Nguyen et al., 2015)
YL-03b	PAV19579	65.6 cM	–	YL-03	44 419 701	–
YL-05c	PAV19079	40.6 cM	–	YL-05	42 794 905	–
YL-05c	CAL0307172.1	–	SPL6-like	YL-05	42 886 478	(Cai et al., 2022)
YL-05c	CAL0307183.1	–	HDA5-like	YL-05	42 951 048	(Luo et al., 2015)
YL-05c	PAV18370	70.7 cM	–	YL-05	43 093 622	–
YL-05c	CAL0307207.1	–	TCP14-like	YL-05	43 101 112	(Lucero et al., 2017)
YL-05c	SNP17629	71.8 cM	–	YL-05	43 154 880	–
YL-13a	SNP15322	6.8 cM	–	YL-13	1 601 669	–
YL-13a	CAL0318261.1	–	TCP14-like	YL-13	10 645 499	(Lucero et al., 2017)
YL-13a	PAV18642	7.5 cM	–	YL-13	10 892 237	–
YL-13c	SNP14194	0.0 cM	–	YL-13	75 230	–
YL-13c	CAL0317433.1	–	NF-YB3-like	YL-13	298 050	(Kumimoto et al., 2008)
YL-13c	PAV18424	9.2 cM	–	YL-13	394 470	–
YL-13c	SNP16639	9.9 cM	–	YL-13	410 673	–
YL-13c	CAL0317462.1	–	SEC13A-like	YL-13	443 719	(Yang et al., 2023)
YL-13c	SNP13118	11.8 cM	–	YL-13	509 385	–
YL-15b	SNP05829	16.6 cM	–	–	–	–
YL-15b	CAL0311711.1	–	ARID5-like	YL-09	2 952 619	(Tan et al., 2020)
YL-15b	CAL0311714.1	–	JMJ32-like	YL-09	2 977 145	(Maruoka et al., 2022)
YL-15b	SNP03753	16.6 cM	–	YL-09	3 029 765	–
YL-16b	PAV19452	0.0 cM	–	–	–	–
YL-16b	CAL0322104.1	–	FRS6-like	YL-16	2 135 034	(Lin and Wang, 2004; Ma and Li, 2018)
YL-16b	CAL0322112.1	–	EMF1-like	YL-16	2 206 530	(Park et al., 2011; Pu et al., 2013)
YL-16b	CAL0322116.1	–	EIL1-like	YL-16	2 239 971	(Xu et al., 2023)
YL-16b	SNP12363	0.4 cM	–	YL-16	2 272 209	–
YL-16b	CAL0322142.1	–	LUX-like	YL-16	2 452 643	(Helfer et al., 2011)
YL-16b	SNP12417	1.9 cM	–	YL-16	2 875 477	–
YL-17	CAL0324083.1	–	MSI4-like	YL-17	34 857 764	(Pazhouhandeh et al., 2011)
YL-17	SNP02609	91.8 cM	–	YL-17	35 004 100	–
YL-17	PAV18230	120.8 cM	–	YL-17	1 523 239	–
YL-17	CAL0323253.1	–	CO-like	YL-17	1 753 510	(Romero et al., 2024)
YL-17	PAV17941	123.1 cM	–	YL-17	1 884 613	–
YL-20a	CAL0326936.1	–	ARID5-like	YL-20	411 124	(Tan et al., 2020)
YL-20a	PAV17700	0.0 cM	–	YL-20	629 038	–
YL-20a	CAL0326974.1	–	SKIP23-like	YL-20	653 511	(Li et al., 2019; Kumar et al., 2022)
YL-20a	SNP17586	11.3 cM	–	YL-20	2 196 200	–
YL-21a	SNP14231	49.9 cM	–	YL-21	26 677 682	–
YL-21a	CAL0328938.1	–	VOZ1-like	YL-21	26 704 878	(Kumar et al., 2018)
YL-21a	CAL0328965.1	–	COL9-like	YL-21	27 032 812	(Cheng and Wang, 2005)
YL-21a	CAL0328975.1	–	ULT1-like	YL-21	27 136 494	(Pu et al., 2013; Xing et al., 2025)
YL-21a	SNP10380	49.9 cM	–	YL-21	27 234 152	–
YL-23a	SNP05931	17.3 cM	–	YL-23	27 940 322	–
YL-23a	CAL0331837.1	–	BLH4-like	YL-23	27 946 875	(Cao et al., 2024)
YL-24	PAV18516	55.8 cM	–	YL-24	28 498 577	–
YL-24	CAL0333067.1	–	WRKY40-like	YL-24	28 877 500	(Li et al., 2026)
YL-24	SNP10516	58.8 cM	–	YL-24	28 893 137	–

Among the unique QTLs, four candidate genes were designated for YL-16b, *FAR1-RELATED SEQUENCE6* (*FRS6*), *EMF1*, *ETHYLENE INSENSITIVE3-LIKE1* (*EIL1*), and *LUX ARRHYTHMO* (*LUX*), while three genes for two QTLs: YL-05c, *SPL6*, *HISTONE DEACETYLASE5* (*HDA5*) and *TCP14*; and YL-21a, *VASCULAR PLANT ONE-ZINC FINGER1* (*VOZ1*), *CONSTANS-like9* (*COL9*), and *ULTRAPETALA1* (*ULT1*). Two candidate genes were chosen for the other two QTLs: YL-15b, *AT-RICH INTERACTING DOMAIN5* (*ARID5*) and *JUMONJI-C-DOMAIN CONTAINING PROTEIN32* (*JMJ32*); and YL-20a, *ARID5* and *SKI-INTERACTING PROTEIN23* (*SKIP23*). Single candidate genes were nominated for the following unique QTLs: YL-02c, *HOOKLESS1* (*HLS1*); YL-03b, *BOI-RELATED GENE3* (*BRG3*); YL-13a, *TCP14*; YL-13c(1), *NUCLEAR FACTOR Y SUBUNIT B3* (*NF-YB3*); YL-13c(2), *SECRETORY13A* (*SEC13A*); YL-17(1), *MULTICOPY SUPPRESSOR OF IRA1.4* (*MSI4*); YL-17(2), *CONSTANS* (*CO*); YL-23a, *BLH4*; YL-24, *WRKY40* (**Table 7**).

## 4 Discussion

### 4.1 Segregation distortion in the RIL mapping population

The linkage map developed in the present study contains several genomic regions that carry markers with significant segregation distortion from the expected 1:1 ratio. According to the basic Mendel’s law of segregation, two alleles at the same locus should be transmitted to offspring with the same probability; however, this rule is violated for so-called segregation distorters, selfish alleles that can spread in the population even if they carry disadvantageous traits (Lyttle, 1991; Ågren and Clark, 2018). Furthermore, inbreeding in a form of selfing promotes the spread of segregation distorters in hermaphroditic plants, and genetic mapping studies have shown an abundance of significantly distorted alleles in highly inbred plant species, including, among others, *Arabidopsis*, rice, barley, pearl millet, and common bean (Xia and Ouyang, 2020; Wang et al., 2024). Large blocks of significantly distorted markers were also reported in two RIL populations developed by the single seed descent method for maize, a typical cross-pollinating species (Pan et al., 2012). Segregation distortion has been commonly encountered legume in linkage mapping, and affected also genetic maps developed for three domesticated Old World lupin species, including also two yellow lupin linkage maps (Phan et al., 2007; Nelson et al., 2010; Książkiewicz et al., 2017; Iqbal et al., 2019; Kozak et al., 2019; Iqbal et al., 2020; Lichtin et al., 2020; Plewiński et al., 2022). Significant segregation distortion has been commonly reported for lupin regions carrying recessive genes for low alkaloid content, and it is explained as a result of sweet-selective insect herbivory during RIL development (Nelson et al., 2006; Iqbal et al., 2019). However, the aforementioned lupin linkage maps contain numerous regions with a skewed segregation of markers that are not associated with alkaloid content. Segregation distortions in mapping populations have been reported to be caused by gametic competition (elimination) or zygotic selection (Li et al., 2011; Baumbach et al., 2012; Reflinur et al., 2014; Dai et al., 2017; Ozer et al., 2025). Interestingly, incorporation of markers with segregation distortion has very little effect on marker order or length when the distances between adjacent markers are relatively large (Hackett and Broadfoot, 2003). In high-density linkage maps, it can even improve the grouping of markers from the same chromosomes and increase the consistency of linkage maps with the genome, as reported for a highly self-pollinating legume, soybean (Zuo et al., 2019). Nevertheless, segregation distortion can have negative implications for genetics and breeding applications, especially when it is based on zygotic selection that affects a large number of loci (Li et al., 2011).

### 4.2 Crosstalk between photoperiod and vernalization pathways at the *LlutFTc1* locus

Our study revealed co-localization of major QTL for all flowering-related phenology traits at the same locus in both photoperiods (short-day and long-day), namely at the PCR marker tagging a large *LlutFTc1* promoter indel sequence. This result provides another line of evidence that the *LlutFTc1* allele conferring vernalization-independent flowering is also associated with significantly reduced response to the photoperiod. This finding provides an explanation for the observed phenological link between photoperiod-responsiveness and vernalization-requirements in yellow lupin; lines which have a strong vernalization requirement become highly responsive to photoperiod if this requirement is not fulfilled (Christiansen and Jørnsgård, 2002). It is also consistent with recent observation that the Parys line carrying the vernalization-responsive *LlutFTc1* allele flowered 20 days later under the short-day photoperiod than under the long-day photoperiod in the absence of vernalization, while the PRH444/14 line carrying the thermoneutral *LlutFTc1* allele showed only 10 days of flowering time difference between photoperiods (Plewiński et al., 2022). Moreover, the PRH444/14 line under the long-day photoperiod was fully thermoneutral in that study. A significant association between *FTc1* alleles and response to vernalization and photoperiod was also evidenced for white lupin and narrow-leafed lupin species (Nelson et al., 2017; Rychel-Bielska et al., 2020, 2024). Rapid flowering of white lupin, narrow-leafed lupin and yellow lupin lines that carry shorter versions of the *FTc1* promoters is supposed to be related to the loss of all binding sites for repressive transcription factors controlling the length of vegetative phase, AGAMOUS-LIKE 15 (AGL15) or SHORT VEGETATIVE PHASE (SVP) (Rychel-Bielska et al., 2020; Plewiński et al., 2022; Surma et al., 2025). Therefore, the observed „crosstalk” between the photoperiod and vernalization pathways at the *FTc1* locus in early lines with indels may result from a widely disrupted repression of this gene in the juvenile phase, rather than from altered signaling of the photoperiod and vernalization pathways. This hypothesis is supported by the detection of high expression of the *FTc1* gene in the early growth stage in narrow-leafed lupin and yellow lupin lines that carry particular *FTc1* indels (Rychel-Bielska et al., 2020; Plewiński et al., 2022), i.e. during the juvenile phase when the photoperiod had no effect on the flowering phenotype (Christiansen et al., 2008). More comprehensive evidence could be provided by expression-QTL mapping during the juvenile phase in both photoperiods and the comparison of the e-QTL peaks with localization of the *FT* genes and the flowering time QTLs.

### 4.3 Photoperiod, autonomous, and vernalization pathways are the most represented among candidate genes

Apart from the major QTL localized at the *FTc1* locus and significant for all trait×year×photoperiod combinations, numerous minor (significant for at least two combinations) and unique (significant for one combination) QTLs were identified, including one specific for under short-day photoperiod. This finding is supported by the previous reports indicating that the *FTc1* polymorphism explains only part of the phenological diversity discovered in lupin species because there is still some variability in flowering time among genotypes carrying the same *FTc1* alleles (Nelson et al., 2017; Plewiński et al., 2020; Taylor et al., 2020; Plewiński et al., 2022; Rychel-Bielska et al., 2024; Surma et al., 2025). The sequence annotation revealed 20 candidate genes for minor QTLs (**Table 6**) and 23 for unique QTLs (**Table 7**) within a sliding window of 250 kb. The 250 kb value represents an initial screening range, selected by comparison of several similar QTL studies targeting, among others, maize yield traits, rice brown spot resistance and salt tolerance, sorghum plant architecture, and wheat kernel weight (Daba et al., 2018; Olatoye et al., 2020; Wang et al., 2022; Zhang et al., 2022; Lin et al., 2025) We anticipate subsequent candidate intervals to be narrowed through more refined mapping or expression analysis. Here, we briefly outline the functions of these genes addressing their assignment to particular pathways.

The most-represented is the photoperiodic pathway that includes the following candidate genes: *bHLH78*, *bHLH93*, *CO*, *EMF1*, *FRS6*, *LUX*, *NF-YB3*, *PRR3*, *TCP7*, *TPR2*, *VOZ1*, and *WRKY40*. bHLH78 binds to chromatin DNA of the *FT* gene and promotes its expression, and thus triggers flowering in response to blue light (Liu et al., 2013). In woodland strawberry bHLH78 binds directly to *FT*, *AGL42*, *LFY*, and *SEP3* to accelerate flowering (Yao et al., 2025). *bHLH93* promotes flowering specifically in short-day conditions in *Arabidopsis* (Sharma et al., 2016; Poirier et al., 2018). *CO* is a major component of the photoperiod pathway (Romero et al., 2024). EMF1 participates in Polycomb group-mediated transcriptional repression of flower the MADS box genes *AGAMOUS*, *APETALA3*, and *PISTILLATA* (Park et al., 2011; Pu et al., 2013; Wang et al., 2014). *FRS6* delays flowering in response to far-red light (Lin and Wang, 2004). *LUX* encodes a night-time repressor of circadian gene expression (Helfer et al., 2011). *NF-YB3* contributes to the promotion of flowering on long days (Kumimoto et al., 2008). *PRR3* regulates the circadian clock and flowering time and is responsive to the photoperiod (Murakami et al., 2004; Li et al., 2020). TCP7 interacts with Nuclear Factor-Ys to promote flowering by directly regulating *SOC1* in *Arabidopsis* (Li et al., 2021) and together with *ARF3* regulates flowering time in chrysanthemum, a short-day plant (Tian et al., 2025). TPR2 delays flowering due to CO degradation (Chang et al., 2019). VOZ1 physically interacts with CO to modulate its function and promote flowering (Kumar et al., 2018). COL9 delays flowering by reducing the expression of *CO* and *FT* (Cheng and Wang, 2005). WRKY40 is involved in the regulation of flowering on long days through transcriptional and epigenetic control of *FT* (Li et al., 2026).

The second frequent is the autonomous pathway, represented here by *LSD1*, *ARF4*, *HDA5*, *EIL1*, *MSI4* (*FVE*), and *ULT1*. LSD1 suppresses the expression of *FWA* and *FLOWERING LOCUS C* (*FLC*) and promotes the floral transition (Jiang et al., 2007). ARF4 regulates flowering through the FVE/MSI4 pathway (Dong et al., 2021, 2022). HDA5 promotes flowering together with *FVE* and *FLD* (Luo et al., 2015). EIL1 modulates *FLC* expression via histone demethylase interaction (Xu et al., 2023). MSI4/FVE is involved in the epigenetic regulation of flowering time by suppressing *FLC* (Pazhouhandeh et al., 2011). ULT1 is a photoperiod-independent regulator of flowering time by H3K27me3-mediated *FLC* repression (Xing et al., 2025).

The third is the vernalization pathway, represented here by *At3g17800*, *HSFB2B*, *SEC13A* and *JMJ32*. *At3g17800* is less recognized than other genes, however, its homolog was found in flowering time QTL in oilseed rape, and was down-regulated in two varieties of rapeseed during low-temperature vernalization (Liu et al., 2022). HSFB2B negatively regulates flowering by fine-tuning the expression of *VERNALIZATION INSENSITIVE3* (*VIN3*) for proper vernalization response (Jeong et al., 2022). SEC13B, an *Arabidopsis* paralog of SEC13A, interacts with the SUPPRESSOR OF FRIGIDA4 (SUF4) to suppress flowering (Yang et al., 2023). JMJ32 modulates the intensity of vernalization by histone demethylation at the *FLC* locus (Maruoka et al., 2022).

Three pathways are represented by two genes each. The age-related pathway is represented by *SPL13A* and *SPL6*, ambient temperature by *bHLH94* and *EFM*, whereas gibberellins by *TCP14* and *BLH4*. *SPL13A* promotes flowering in lilly and cucumber (Yamagishi et al., 2024; Ye et al., 2024), while *SPL6* accelerates flowering in switchgrass, putatively via miR156 age-related regulation (Cai et al., 2022). *bHLH94* mediates the development of axillary buds and spike initiation in *Phalaenopsis aphrodite* at 20 °C (Lin et al., 2019), whereas *EFM* mediates flowering responses to ambient temperature (but not vernalization) and light (but not photoperiod) in *Arabidopsis* (Yan et al., 2014). *TCP14* probably modulates *SOC1*-dependent flowering, acting downstream of gibberellins (Resentini et al., 2015; Lucero et al., 2017), while *BLH4* accelerates heading date in rice by gibberellin activation (Cao et al., 2024).

In addition to pathway-specific components, candidate genes include some pathway integrators and basic regulatory genes, such as *ARID5*, *BRG3*, *FTIP1*, *HARBI1*, *HLS1*, *LFY*, *SPL1*, and *SWC4*. ARID5 is a component of the chromatin-remodeling complex and regulates floral transition by dual recognition of AT-rich DNA and H3K4me3 (Tan et al., 2020). BRG3 inhibits *FT* expression by targeting the *FT* locus in the presence of DELLAs through a CO-dependent and CO-independent mechanism (Nguyen et al., 2015). *FTIP1* is an essential regulator required for florigen transport (Liu et al., 2012; Fang et al., 2024). *HLS1* regulates pleiotropic developmental processes and delays flowering time (Wang et al., 2023; Nobusawa et al., 2025) LFY activates floral homeotic genes (Parcy et al., 1998; Wagner et al., 1999). HARBI1 regulates *Pugionium cornutum* flowering and seed development through down-regulation of *FLC* and activation of *FT* and *SOC1* (Zhao et al., 2026). *SPL*, together with *SOC1*, integrates the photoperiod and gibberellic acid pathways (Jung et al., 2012). In fact, SPL1-like in *Antirrhinum majus* initiates flowering through the activation of meristem identity genes (Preston and Hileman, 2010). SWC4 modulates histone H2A.Z deposition, repressing the transcription of a number of genes, including the floral integrator *FT* and key transcription factors (Gómez-Zambrano et al., 2018).

The remaining three candidate genes, *ADA2b*, *ERF114*, and *SKIP23* are not yet assigned to any of the pathways. *ADA2b* mutants have delayed the transition from juvenile to adult phase and are late flowering (Kim et al., 2015; Wu et al., 2021). *ERF114*, an ethylene responsive factor, regulates the floral transition in *Catalpa bungei* (Fei et al., 2025). *SKIP23* just co-localized with one of a few SNPs identified by the principal component analysis as associated with flowering in pigeonpea (Kumar et al., 2022).

### 4.4 Potential sources of inter-annual phenotypic variability

We observed significant advance of plant phenology in 2024 as compared to 2022 and 2023 (**Supplementary Table S4**). To evaluate potential source of this variability we accessed publicly available meteorological data on daily mean cloud cover, sunshine hours and maximum temperature recorded at the closely-located (6 km away from greenhouse) SYNOP meteorological station Poznań-Ławica (https://danepubliczne.imgw.pl/). The analysis of these data revealed that during the first eight weeks of plant growth in 2024 the daily number sunshine hours and the daily maximum temperature were higher than in the corresponding periods in 2022 and 2023 (**Table 8**), however, observed differences were significant only for the maximum temperature (p-values for 2022 vs 2023 comparison is 0.996, for 2022 vs 2024 is 0.046 and for 2023 vs 2024 is 0.037). In general, warm temperature accelerates flowering by FLOWERING LOCUS M (FLM)-mediated SVP degradation (Xuan et al., 2022). As the final target of this regulatory mechanism is the promoter of *FT* gene (Lee et al., 2017), it may be not possible to elucidate QTL effect for this pathway in Parys×PRH444/14 mapping population due to the presence of large indel in the *LlutFTc1* promoter carrying many potential binding sites for transcription factors. Given the increasing knowledge on the complexity of high temperature regulation of flowering time in model and non-model plant species (Luo et al., 2025), the contribution of other genetic components cannot be ruled out.

**Table 8:** Mean values of selected meteorological conditions recorded at Poznań-Ławica SYNOP meteorological station.

Year	2022	2023	2024	2022	2023	2024	2022	2023	2024
Week	Cloud cover	Cloud cover	Cloud cover	Sunshine hours	Sunshine hours	Sunshine hours	Maximum temperature	Maximum temperature	Maximum temperature
1	4.1	6.8	6.4	6.5	3.0	6.0	11.5	14.0	19.0
2	0.9	5.9	6.7	9.4	4.3	6.8	15.3	11.2	21.0
3	5.6	5.1	6.1	6.7	4.7	6.1	9.9	8.3	17.9
4	6.3	6.6	6.5	3.7	3.6	1.9	10.8	12.2	8.0
5	5.0	7.1	5.2	7.5	2.1	9.0	14.6	15.0	18.7
6	5.4	4.1	4.0	5.1	7.0	10.7	13.4	16.1	23.7
7	4.7	4.4	3.2	6.3	9.9	10.8	16.8	16.9	19.9
8	4.1	3.4	3.3	7.8	10.4	10.8	19.9	17.1	24.1
Mean	4.5	5.4	5.2	6.6	5.6	7.8	14.0	13.9	19.0

### 4.5 Conclusions

The present study reports on the development of a yellow lupin linkage map, which was constructed for a newly established mapping population that descends from a rapid flowering PRH444/14 breeding line, independent of vernalization and non-responsive to photoperiod. Alignment of this linkage map with the recently released yellow lupin genome sequence revealed a high level of collinearity, positively validating the assembly of particular pseudochromosomes. The QTL mapping of the yellow lupin flowering phenology traits confirmed the main role of the *LlutFTc1* gene in the responses to vernalization and photoperiod, while highlighting several new QLTs, including one specific for a short day photoperiod. Sequence annotation revealed that almost all reported QTLs carry potential candidate genes from the flowering regulatory network, predominantly acting in photoperiod, autonomous, and vernalization pathways.

## Data Availability

The original contributions presented in the study are included in the article/**Supplementary Material**. Further inquiries can be directed to the corresponding author.

## Supplementary material

Supplementary Table S1 Results of yellow lupin phenotyping for flowering phenology traits.

Supplementary Table S2 Mean values of yellow lupin phenotypic observations of flowering phenology traits.

Supplementary Table S3 PCR-based markers developed for yellow lupin linkage mapping.

Supplementary Table S4 Results of inter-annual comparisons by Kruskal Wallis test by ranks with *post-hoc* Dunn’s test.

Supplementary Table S5 Wilcoxon signed rank test pairwise comparison of parental lines with RILs showing the most extreme phenotypes.

Supplementary Table S6 Results of DNA isolation from yellow lupin RIL lines.

Supplementary Table S7 Sequence-based markers localized on the yellow lupin linkage map.

Supplementary Table S8 Segregation data for markers localized on the yellow lupin linkage map.

Supplementary Table S9 QTLs identified for yellow lupin flowering phenology traits using 3–10 cM window size and 5–25 background markers.

Supplementary Table S10 Results of QTL mapping of yellow lupin flowering phenology traits for 10 cM window size and 15 background markers.

Supplementary Table S11 Spearman’s rank correlation coefficient calculated for yellow lupin flowering phenology traits and segregation data of 1448 non-redundant loci among 136 RILs from yellow lupin mapping population.
